# Persistent DNA damage associated with ATM kinase deficiency promotes microglial dysfunction

**DOI:** 10.1093/nar/gkac104

**Published:** 2022-02-25

**Authors:** Julie Bourseguin, Wen Cheng, Emily Talbot, Liana Hardy, Jenny Lai, Ailsa M Jeffries, Michael A Lodato, Eunjung Alice Lee, Svetlana V Khoronenkova

**Affiliations:** Department of Biochemistry, University of Cambridge, 80 Tennis Court road, CambridgeCB2 1GA, UK; Department of Biochemistry, University of Cambridge, 80 Tennis Court road, CambridgeCB2 1GA, UK; Department of Biochemistry, University of Cambridge, 80 Tennis Court road, CambridgeCB2 1GA, UK; Department of Biochemistry, University of Cambridge, 80 Tennis Court road, CambridgeCB2 1GA, UK; Division of Genetics and Genomics, Boston Children's Hospital; Department of Pediatrics, Harvard Medical School, Boston, MA 02215, USA; Broad Institute of MIT and Harvard, Cambridge, MA 02142, USA; Program in Neuroscience, Harvard University, Boston, MA 02115, USA; Department of Molecular, Cell, and Cancer Biology, University of Massachusetts Medical School, Worcester, MA 01605, USA; Department of Molecular, Cell, and Cancer Biology, University of Massachusetts Medical School, Worcester, MA 01605, USA; Division of Genetics and Genomics, Boston Children's Hospital; Department of Pediatrics, Harvard Medical School, Boston, MA 02215, USA; Broad Institute of MIT and Harvard, Cambridge, MA 02142, USA; Department of Biochemistry, University of Cambridge, 80 Tennis Court road, CambridgeCB2 1GA, UK

## Abstract

The autosomal recessive genome instability disorder Ataxia–telangiectasia, caused by mutations in ATM kinase, is characterized by the progressive loss of cerebellar neurons. We find that DNA damage associated with ATM loss results in dysfunctional behaviour of human microglia, immune cells of the central nervous system. Microglial dysfunction is mediated by the pro-inflammatory RELB/p52 non-canonical NF-κB transcriptional pathway and leads to excessive phagocytic clearance of neuronal material. Activation of the RELB/p52 pathway in ATM-deficient microglia is driven by persistent DNA damage and is dependent on the NIK kinase. Activation of non-canonical NF-κB signalling is also observed in cerebellar microglia of individuals with Ataxia–telangiectasia. These results provide insights into the underlying mechanisms of aberrant microglial behaviour in ATM deficiency, potentially contributing to neurodegeneration in Ataxia–telangiectasia.

## INTRODUCTION

Ataxia–telangiectasia (A–T) is an autosomal recessive disease caused by loss-of-function mutations in the *ATM* gene (A–T mutated) (1). ATM is a serine/threonine protein kinase that plays a central role in coordinating the cellular response to genotoxic stress, in particular cytotoxic and mutagenic DNA double-strand breaks (2,3). Consequently, cellular processes regulated in an ATM-dependent manner include chromatin decondensation, apoptosis, senescence, cell cycle, redox balance, metabolism and splicing (4).

A–T has a wide range of clinical manifestations, however, one of the most devastating clinical signs of the classical form of A–T is neurodegeneration. The disease manifests as motor dysfunction in young children and is predominantly characterized by the progressive loss of Purkinje and granule neurons in the cerebellum (5). Cerebellar degeneration is thought to be linked with defects in the neuronal DNA damage response, metabolic abnormalities, and epigenetic silencing of diverse neuronal genes (4,5). However, the molecular mechanisms that underpin cerebellar degeneration in A–T are poorly understood.

Microglia are resident immune cells of the central nervous system (CNS). Microglia play instrumental roles in the development and maintenance of the CNS, including regulation of neurogenesis, synaptic maintenance and plasticity, trophic support of other cell types, and clearance of apoptotic and dead cells. However, persistent microglial activation and consequent neuroinflammation are implicated in the pathology of diverse neurodegenerative disorders (6). For example, somatic mutations in the BRAF oncogene in erythro-myeloid precursors of microglia result in progressive neurological impairment with multiple features of cerebellar ataxia (7). In addition, microglial priming (an enhanced response to secondary stimuli) and activation, which are both linked to neuroinflammation, are seen in mouse models of *Ercc1^Δ^^/–^*-linked nucleotide excision repair and frataxin deficiencies (8,9).

In Atm-deficient mice and rats, morphological changes associated with microglial activation have been observed, and microglia were shown to mount a neurotoxic innate immune response to cytosolic DNA (10–13). Pharmacological inhibition of Atm in cultured *ex vivo* murine microglia led to neuronal cell death, mediated by the secretion of the neurotoxic cytokine IL-1β, which was dependent on the cytosolic DNA sensing adaptor STING (12). In addition, links between ATM deficiency and activation of the innate immune signalling have been demonstrated in other cell types (14–17). Rodent models of ATM deficiency, however, do not fully recapitulate the human phenotype, possibly due to a higher sensitivity of the human CNS to oxidative stress and DNA damage (11,18). Moreover, microglia from rodents and humans show fundamental differences in their response to extracellular signalling molecules and secretion, and exhibit highly divergent gene expression signatures (19–21).

In the present work, we utilized human cell models to investigate the underlying basis for the role of microglia in neurodegeneration in A–T. We find that loss of ATM, or its activity, promotes sustained microglial activation linked with increased expression of pro-inflammatory cytokines and phagocytic clearance. Microglial activation was shown to be mediated by the non-canonical RELB/p52 nuclear factor NF-κB transcriptional pathway. The RELB/p52 pathway is activated in response to persistent DNA damage associated with A–T and is driven by the NF-κB-inducing NIK kinase. Chronic activation of ATM-deficient microglia results in excessive phagocytosis of neurites, potentially contributing to neurodegeneration. Analyses of single-nucleus RNA-sequencing data identified activation of non-canonical NF-κB signalling in cerebellar microglia from individuals with A–T versus healthy controls, highlighting the relevance of our findings to human disease. These data provide mechanistic insights into microglial dysfunction in human A–T.

## MATERIALS AND METHODS

### Reagents and materials

Reagents and materials used in this work are described in Supplementary Table S1.

### Cell culture

Immortalized human fetal microglial HMC3 cells were established by Marc Tardieu and kindly provided by Brian Bigger (University of Manchester) (22). HMC3 cells were grown in DMEM (Gibco; 4.5 g/l glucose, no pyruvate) supplemented with 10% heat-inactivated fetal bovine serum (HI-FBS; Merck). Immortalized human C20 microglia were kindly provided by David Alvarez-Carbonell (23). C20 cells were grown in DMEM:F12 (Lonza) supplemented with 1% N-2 (ThermoFisher) and 1% HI-FBS. Microglia were routinely seeded at the density of 3 000–6 000 cells/cm^2^ reaching 70–80% confluency at the end of an experiment.

Fetal mesencephalic LUHMES neuronal progenitors were purchased from ATCC (CRL2927) (24). Cells were grown in DMEM:F12 supplemented with 1% N-2 and 40 ng/ml recombinant zebrafish basic FGF (Hyvönen laboratory, Department of Biochemistry, University of Cambridge) in vessels coated with 50 μg/mL poly-l-ornithine (Merck) and 1 μg/ml human fibronectin (Millipore). Cells were seeded at a density of 35 000–50 000 cells/cm^2^ and differentiated for 5 days in DMEM:F12 medium supplemented with 1% N-2, 0.25 mM dibutyryl-cAMP (SelleckChem), 1 μg/mL tetracycline (Merck) and 2 ng/ml recombinant human GDNF (R&D Systems).

All cells were cultured at 5% CO_2_, 37°C and 95% humidity. Cell lines were routinely tested for viability and mycoplasma, and kept in culture for no longer than 40–50 population doublings.

### Establishment of knockout and rescue cell lines

*ATM* KO HMC3 cells were generated using a vector encoding SpCas9(D10A) nickase (kind gift of Feng Zhang; Addgene plasmid #48141) (25) and sgRNAs targeting *ATM* exon 4:

sense_sgRNA: GATGCAGGAAATCAGTAGTT

anti-sense_sgRNA: TGTGTTGAGGCTGATACATT

HMC3 cells were transfected with the vector using Lipofectamine 2000 (ThermoFisher), selected with 1 μg/ml puromycin for 5 days and single-cell cloned. Similar approach was used to generate *ATM* KO LUHMES neuronal lines, except the pSpCas9(BB)-2A-Puro V2.0 vector with improved puromycin selection (kind gift of Feng Zhang; Addgene plasmid #62988) (25) and the sgRNA targeting exon 6 of *ATM* (CCAAGGCTATTCAGTGTGCG) were used. *ATM* knockout C20 lines were generated using the AIO-GFP vector encoding GFP-tagged SpCas9(D10A) nickase (kind gift of Steve Jackson; Addgene plasmid #74119) (26) and sgRNAs targeting *ATM* exon 4 as above. After transfection, GFP-positive C20 microglia were sorted as single cells using the MoFlo XDP Cell Sorter (Beckman-Coulter). Successful *ATM* KO clones were identified by fragment analysis using capillary electrophoresis (Applied Biosystems) and/or immunoblotting. Gene editing was confirmed by Sanger sequencing.

Re-expression of ATM in *ATM* KO HMC3 cells was achieved using the piggyBac transgene carrying FLAG-tagged wild-type *ATM* gene. *ATM* was PCR amplified using Phusion high-Fidelity DNA polymerase (ThermoFisher) from the pcDNA3.1 Flag-His-ATM plasmid (kind gift of Michael Kastan; Addgene plasmid #31985) (27) as two individual fragments with 20-nt overhangs. The oligonucleotide sequences are shown in Supplementary Table S1 (overhangs in low case, DNA sequences complementary to the gene sequence are capitalized). The piggyBac backbone was prepared by a NotI-XhoI restriction digest using the pPB-TRE IRES-mCherry plasmid (kind gift of Bon-Kyoung Koo) (28). The fragments were assembled using NEBuilder HiFi DNA Assembly Cloning kit (New England Biolabs) to yield the pPB-TRE Flag-wtATM plasmid, and confirmed by Sanger sequencing. *ATM* KO HMC3 cells were transfected with pPB-TRE Flag-wtATM, rtTA and transposase encoding plasmids (28) at a 3:3:1 ratio using the Glial-Mag Magnetofection kit according to the manufacturer's protocol (OzBiosciences). The cells were selected using 250 ng/ml hygromycin B for 7 days and maintained as a polyclonal population in standard growth medium supplemented with 50 ng/ml of hygromycin B. Expression of ATM was induced using 25–100 ng/ml doxycycline.

### RNA interference

Cells were transfected with 50 nM final siRNA for 72 h using Lipofectamine RNAiMax according to the manufacturer's guidelines (Thermo Fisher Scientific). siRNA Oligonucleotides were synthesized by Merck (sequences are given in the Supplementary Table S1).

### Cell treatments

To induce NF-κB signalling, human recombinant TNFα (PeproTech) was used at 10 ng/ml for 0.5-6 h. ATM inhibitor AZD1390 (SelleckChem) was used at a final concentration of 10 nM for at least 1 h and up to 9 days (DMSO served as a control) in HMC3 cells and at 100 nM in LUHMES cells during days 2–5 of differentiation. For long-term inhibitor treatments, the medium with inhibitor was renewed every 48 h. For the inhibitor wash-out, cells were washed with PBS and cultured in the absence of inhibitor for an additional 2 days. To induce ATM-dependent phosphorylation, cells were treated with 1 μM camptothecin (CPT; Cambridge Bioscience) for 1 h. To induce apoptosis, cells were treated with 0.33 μM staurosporine (Merck) for 16 h. Treatment with 2 μM hydrogen peroxide for 5 min (H_2_O_2_; Merck) was used to induce DNA strand-breaks and oxidative damage. To induce persistent DNA damage, cells were treated with 0.5 μM etoposide, or DMSO as a control, or with 100–250 μM *tert*-butyl hydroperoxide (tBHP) for up to 6 days (Merck). The medium with tBHP or etoposide was renewed every 24 or 48 h, respectively. A scavenger of superoxide radicals, Tiron (Merck), was used at 10 μM over a 72-h period, and the medium containing the compound was changed daily.

### Protein extraction, biochemical fractionations and immunoblotting

Whole-cell, cytoplasmic and nuclear extracts were prepared as previously described (29,30). Proteins were separated by 4–16% Tris-glycine SDS-PAGE, transferred onto PVDF membranes and the membranes were blocked in Intercept–TBS buffer (LI-COR Biosciences). Primary antibodies were diluted in Intercept–TBS, 0.1% Tween-20. Fluorescent secondary antibodies were used at 1:20 000 and diluted in Intercept-TBS, 0.1% Tween-20, 0.01% SDS. The antibodies are described in the Supplementary Table S1. Membranes were imaged using an Odyssey CLx system and quantified using Image Studio (LI-COR Biosciences). Quantifications were carried out relative to loading controls or, in case of phosphorylated proteins, to both the total proteins and loading control. Levels of ATM^pS1981^ were quantified without regard to total protein since recognition of the total protein by the antibody is dependent upon its phosphorylation status. In the case of some antigens, such as NIK, the quantification was not feasible due to low levels of detection. Immunoblot images that are representative of at least three independent biological experiments are shown in figures.

### Quantitative RT-PCR

RNA was extracted using an RNeasy Mini Kit as per the manufacturer's instructions (QIAGEN). RNA integrity was verified on agarose gels by assessing the 28S:18S rRNA ratio. RNA was treated with DNAse I (ThermoFisher) and reverse transcribed using qPCRBIO cDNA Synthesis Kit (PCR biosystems) according to the manufacturer's instructions. For oligonucleotide sequences refer to the Supplementary Table S1. RT-qPCR was performed using qPCRBIO SyGreen Blue Mix Lo-ROX (PCR biosystems) and the QuantStudio 5 Real Time PCR system (ThermoFisher). The data were analysed using QuantStudio Design and Analysis software (ThermoFisher). The following cycling program was used: initial denaturation and polymerase activation at 95°C for 2 min; 40 cycles with 5 s at 95°C followed by 30 s at 65°C; followed by a melting curve step (15 s at 95°C; 1 min at 60 C; 15 s at 95°C). Cycle threshold (Ct) values for all mRNAs of interest were determined for three technical replicates per sample, normalized to RPS13 or IPO8 reference mRNA, and the data were expressed as fold-change relative to the control using the 2^–ΔΔCt^ method.

### Comet (alkaline single cell electrophoresis) assays

Suspension cells were left untreated or treated with 2 μM hydrogen peroxide for 5 min on ice (positive control) and embedded in low-melting point agarose for 2 min on a microscope slide. The cells were lysed in buffer containing 10 mM Tris–HCl, pH 10.5, 2.5 M NaCl, 100 mM EDTA, 1% (v/v) DMSO and 1% (v/v) Triton X-100 for 1 h at 4°C. To unwind the DNA, slides were incubated in cold electrophoresis buffer containing 300 mM NaOH, pH 13, 1 mM EDTA, 1% (v/v) DMSO for 30 min in the dark and electrophoresed at 1.2 V/cm for 25 min. The samples were neutralized with 0.5 M Tris–HCl, pH 8.0 and stained with SYBR Gold nucleic acid stain (Molecular Probes) for 30 min. Samples were analysed in a blinded fashion. Percentage of tail DNA was determined for two technical replicates per sample with at least 50 cells per replicate using the CASP software (31).

### Proliferation assays

For proliferation assays, cells were labelled with 5 μM carboxyfluorescein succinimidyl ester (CFSE; Tonbo Biosciences) for 8 min at 37°C and seeded at progressively reduced density (no less than 3 000 cells/cm^2^). After recovery for 24 h, samples were collected at 24-h intervals and fixed in 70% ice-cold ethanol. CFSE fluorescence intensity was recorded for 10 000 single-cell events using a BDAccuri C6 flow cytometer (BD Biosciences). The mean of fluorescence intensity (MFI) of the green FL1-A channel (excitation 488 nm, emission 530 ± 15 nm) was calculated using FlowJo software. Relative growth for each time point was calculated by normalizing MFI^–1^ of individual time points to the MFI^–1^ value at 24 h post-seeding.

### Cell viability assays

Cells were grown in opaque-walled 96-well plates in the presence of 5–100 nM of camptothecin, or DMSO as control, for 72 h. Technical triplicates were used per condition. Cell viability was determined using CellTiter-Glo 2.0 (Promega) according to the manufacturer's protocol. Luminescence intensity was measured using PHERAstar FS plate reader (BMG Labtech). Surviving fractions for each concentration of the drug were calculated relative to DMSO control.

### Detection of ROS and CD40 levels by flow cytometry

To detect intracellular ROS, adherent cells were incubated with 2.5 μM H_2_DCF-DA (Molecular Probes) for 30 min. Cells were washed with PBS, collected by trypsinization and analysed by flow cytometry on the green FL-1A channel.

For cell surface staining of CD40, live cells were incubated with anti-CD40 antibodies (GeneTex, GTX14148) for 60 min on ice, counterstained with Alexa 488 goat anti-mouse secondary antibody and analysed by flow cytometry on the green FL-1A channel. Relative intracellular ROS content and CD40 expression were calculated by normalizing MFI of each sample to the MFI value of the control.

### Apoptosis assays

Adherent cells were collected by trypsinization (cell growth medium and any PBS washes were collected and combined with cells), stained with Annexin V-FITC and propidium iodide (PI), according to the manufacturer's protocol (Abcam), and analysed by flow cytometry on the green FL-1A and red FL-2A channels. Cells positive for Annexin V, double positive for Annexin V and PI, and positive for PI only were considered as apoptotic, late apoptotic and necrotic, respectively.

### Transwell assays

HMC3 cells were seeded onto transwell membrane inserts with a pore diameter of 0.4 μm (Greiner) and allowed to attach for 24 h. The transwell inserts with HMC3 microglia were transferred into the wells with freshly differentiated post-mitotic LUHMES neurons that were pre-treated with 100 nM AZD1390 and cultured in 0.5% HI-FBS-DMEM or LUHMES differentiation medium supplemented with 1% HI-FBS for 48 h. The number of microglia was controlled by collecting the cells from transwell inserts and counting. Neuronal health was analysed by immunofluorescence. Treatment of LUHMES cells with 0.33 μM staurosporine for 16 h served as positive control for apoptosis.

### Microglial-neuronal co-culture assays

HMC3 cells were labelled with 5 μM CFSE for 8 min at 37°C. Post-mitotic LUHMES cells were labelled with 5 μM CellTrace Violet (CTV; ThermoFisher) according to the manufacturer's protocol. At 24 h post-labelling HMC3 microglia were trypsinized, checked for viability and seeded onto post-mitotic LUHMES neurons at a ratio of 1:5. The co-cultures were maintained in 0.5% HI-FBS-DMEM or differentiation medium supplemented with 1% HI-FBS for 24 h followed by their analyses by flow cytometry and immunofluorescence.

### Phagocytosis assays

At 24 h prior to phagocytosis assays, the growth medium on HMC3 microglia was replaced with 0.5% HI-FBS DMEM. Fluorescent carboxylated 5 μm beads (Spherotech) were added to cells at a ratio of 5:1 and incubated for 6 h at 37°C, 5% CO_2_ and 95% humidity. As a negative control, the assays were carried out in the presence of 1 μM Cytochalasin D. Cells were washed 5 times with ice-cold PBS, collected in 2% HI-FBS/PBS on ice and 10 000 single-cell events were acquired as above. The percentage of cells, which engulfed beads, was determined on the green FL-1A channel after subtracting the percentage of cytochalasin D controls (excitation 488 nm, emission 530 ± 15 nm). Phagocytic activity was calculated by normalizing MFI of all cells to MFI of a single bead.

Microglial phagocytosis of post-mitotic neurons was carried out in microglial-neuronal co-cultures as above. The cultures were washed with ice-cold 0.25 mM EDTA/PBS and collected by trypsinization using 2% HI-FBS/PBS. Individual cell populations were gated and 10 000 events corresponding to CFSE-positive microglia were acquired on the green channel as above using an AttuneNxt flow cytometer (ThermoFisher). The percentage of phagocytic cells was calculated by identifying cells, which were positive for CTV and CFSE, on the violet channel (excitation 405, emission 450 ± 40 nm).

### Analysis of microglial morphology

Automated analyses of microglial morphology in co-cultures with post-mitotic LUHMES neurons were performed using CellProfiler software version 4.0.7 (32). Firstly, nuclei were segmented independent of cell type based on DAPI staining. Microglial cells were then defined as secondary objects with CFSE staining above a specific intensity threshold. Circularity (termed ‘form factor’) of CFSE-positive microglia was calculated as 4π[area]/[perimeter]^2^ where a circularity of 1 defines a perfect circle.

### Immunofluorescence

Cells grown on glass coverslips were fixed in 4% methanol-free paraformaldehyde for 15 min and permeabilized in 0.2% Triton-X100/PBS for 10 min. Coverslips were blocked in 3% BSA, 0.05% Tween-20/PBS, incubated with primary antibodies for 16 h at 4°C and further incubated with fluorescently labelled secondary antibodies (1:500 dilution). Coverslips were mounted with the mounting medium (DAKO) containing 1.5 μg/ml DAPI. The antibodies are described in the Supplementary Table S1.

In all immunofluorescent analyses, images of at least five fields of view (field of view: 318 × 318 μm) per coverslip with at least a total of 100 cells were taken in a blinded manner using an epi-fluorescent Zeiss Axio Observer Z1 (images in a single Z-plane) or a confocal Nikon Eclipse Ti (compressed Z-stack images) microscope. Confocal imaging was sequential for different fluorophore channels to obtain a series of axial images. A secondary antibody only control was used to subtract the non-specific background during quantifications. Analyses were performed using ImageJ and CellProfiler.

To determine the levels of CD68, integrated intensity was measured using compressed Z-stack images.

The percentage of cells containing nuclear p65 or RELB staining was determined using nuclear (defined using DAPI) versus cytoplasmic (defined using vinculin) MFI. The analyses were automated and performed using CellProfiler software (32) and ImageJ.

Phagocytic events in microglial-neuronal co-cultures were identified using compressed Z-stack confocal images and XZ/YZ orthogonal projections. Phagosome-like structures, which were characterized by the disruption of microglial CFSE staining, containing neuronal material were quantified in a randomly acquired tile of 7 × 5 fields of view (2194 × 1567 μm). Internalization events positive for both cleaved caspase-3 and CTV were considered as the uptake of apoptotic neurons, whereas events negative for cleaved caspase-3 and positive for CTV included phagocytosis of healthy cells and/or their parts. Additionally, events positive for β3-tubulin and DAPI represented the uptake of neuronal soma, whereas events positive for β3-tubulin and negative for DAPI were considered as the uptake of neurites. The relative frequency of these events was quantified.

Microglial clusters were quantified in co-culture images using CellProfiler (32). Briefly, clusters of CFSE-positive microglia were selected from other objects using automated pre-defined parameters (size > 4 000 μm^2^; minimum of five proximal cells) and quantified. The cluster areas were further expanded by including 20-μm border pixels to define cluster regions of interest (ROIs). To calculate the events of colocalization of microglial clusters and the damaged neuronal network, neurite network analysis was carried out using β3-tubulin-stained images of neurons in co-culture in cluster ROIs. Non-cluster regions in co-cultures and neuronal only cultures served as controls. The neuronal network in ROIs was masked, skeletonized and the median neurite length normalized to the ROI area was measured. Cluster ROIs, in which a reduction in the area-normalized median neurite length by at least 50% as compared to non-cluster regions/neuronal only cultures was observed, were considered as associated with damage to the neuronal network and quantified.

### Gene set enrichment analyses in human single-nucleus RNA-sequencing data

Human microglia single-nucleus RNA-sequencing data from A–T and control cerebellum were obtained as described (33). Canonical and non-canonical NF-κB pathway gene sets were curated from the literature (34,35). Non-canonical NF-κB targets were obtained from (36) (Supplementary Table S2). To determine the enrichment of each gene set, we implemented a *t-*score permutation test following a statistical approach (37). *T-*scores for each gene were obtained from the differential gene expression analysis of A–T compared to control cerebellar microglia (33). Briefly, the analysis was performed using edgeR. The read counts were modelled and normalized using a negative binomial distribution with the trimmed mean of M-values (TMM) normalization method. The design matrix formula was ∼ disease.status + sex + age + cellular detection rate. Differentially expressed genes between A–T and control were identified using the likelihood ratio test (glmLRT). Genes with large changes in gene expression and small variance across cells will have a large *t*-score value, with the sign of the *t-*score determined by the direction of the fold change in gene expression. For each gene set, the average *t-*score was calculated by averaging the *t*-scores of the genes in the gene set (observed gene set average *t-*score). To generate a distribution of gene set average *t-*scores under the null hypothesis, we calculated the average *t-*score of a randomly sampled equal size gene set with *n* = 10 000 permutations. An exact one-sided *P-*value (upper-tail) was obtained by comparing the observed gene set test statistics with the null distribution. Gene sets with Bonferroni corrected *P-*values < 0.05 were considered significantly upregulated.

### Quantification and statistical analysis

Quantitative data are expressed as the mean of at least three independent biological experiments ± standard deviation (SD) unless stated otherwise. The data were tested for normality using the Shapiro Wilk test and the analysis informed the choice of parametric or non-parametric statistical tests. When normalization of data to the respective control was carried out, a one-sample *t-*test with a theoretical mean of 1 was used. For comparison of two samples, paired or unpaired two-tailed *t-*tests were used with Welch's correction for unequal variances. To compare three or more unmatched samples, a one- or two-way ANOVA was used. The comparison of data obtained from different fields in immunofluorescence was carried out using a non-parametric Wilcoxon test (paired samples – variables counted from the same field). The exact *P*-values are indicated in figure legends, and were considered to be statistically significant when *P* < 0.05. The analyses were performed using GraphPad Prism.

## RESULTS

### Loss of ATM function results in microglial activation

**Figure 1.**
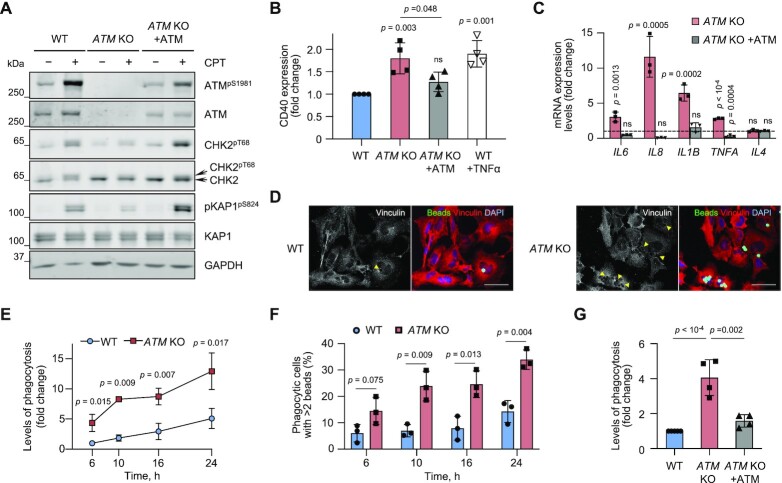
*ATM*-knockout (KO) microglia are activated, resulting in increased expression of pro-inflammatory cytokines and enhanced phagocytic clearance. (**A**) Representative immunoblot analysis of WT, *ATM* KO and *ATM* KO cells in which ATM was re-expressed from a piggyBac transgene. To test for ATM activity, cells were treated with 1 μM camptothecin (CPT) for 1 h. Loading control: GAPDH. (**B**) Cell surface expression levels of the microglial activation marker, CD40, measured by flow cytometry in cells as in (A). Expression is relative to WT HMC3. Positive control: 10 μg/ml TNFα for 6 h, cells collected for analysis at 20 h post-treatment. Mean ± SD shown (*n* = 4). (**C**) RT-qPCR analysis of the indicated cytokines in cells as in (A). Expression is relative to WT HMC3 (dashed line). Reference genes: *RSP13* and *IPO8*. Mean ± S.D. shown (*n* = 3). One-way ANOVA with Dunnett's multiple comparison's test used. (**D**) Representative immunofluorescence images of phagocytic uptake of 5 μm beads in WT and *ATM* KO HMC3 (6 h assay). Confocal Z-stack compression images shown. Arrowheads indicate disruption in vinculin structure associated with bead engulfment. Scale bar: 50 μm. Bead substrates (green), vinculin (red), DNA (blue). (**E**) Kinetics of phagocytosis (5 μm beads) in WT and *ATM* KO cells at the indicated time points. Phagocytosis is relative to WT HMC3 at 6 h with 3.7 ± 1.3% of phagocytic cells. Mean ± S.D. shown (*n* = 3). (**F**) Kinetics of the changes in percentage of highly phagocytic cells (>2 beads per cell) as in (E). To determine the number of beads per cell, Mean fluorescence intensity (MFI) of the population of phagocytic cells was divided by MFI of a single 5 μm fluorescent bead. Mean ± S.D. shown (*n* = 3). Unpaired two-tailed *t*-test used. (**G**) Phagocytosis levels (6 h assay) of cells as in (A). Phagocytosis is relative to WT HMC3, in which 5.8 ± 3.2% of cells are phagocytic. Mean ± S.D. shown (*n* = 4). (B, E, G) One-way ANOVA with Tukey's multiple comparison's test used.

The effects of ATM deficiency on microglial function were studied in immortalized HMC3 and C20 cell lines derived from human microglia (22,23,38). To provide an *ATM*-deficient model system that is representative of A–T (39), an *ATM* knockout (KO) HMC3 cell line was generated using CRISPR/Cas9 (Supplementary Figures S1A and B). The loss of ATM function was verified by treating *ATM* KO microglia with camptothecin, a topoisomerase I inhibitor that induces ATM activation (40). As expected, *ATM* KO microglia displayed: (i) no visible autophosphorylation of ATM at S1981 and grossly attenuated phosphorylation of ATM’s downstream targets, CHK2 and KAP1, in response to camptothecin (Figure 1A; Supplementary Figure S1C; note that KAP1 could also be a substrate for DNA-PKcs in the absence of ATM, hence residual phosphorylation) (41–44), (ii) reduced cell proliferation (Supplementary Figure S1D) and (iii) increased sensitivity to camptothecin compared to wild-type (WT) cells (Supplementary Figure S1E).

To determine whether the *ATM* KO microglia were activated, we first measured expression levels of two markers of microglial activation, CD40 and CD68 (45,46). Cell surface levels of CD40 were increased in *ATM* KO microglia to a similar extent as in WT cells stimulated with TNFα (Figure 1B). Additionally, the levels of lysosomal CD68 were higher in *ATM* KO microglia as compared to WT (Supplementary Figure S2A). To further investigate the activation status of *ATM* KO microglia, we measured mRNA expression of the pro-inflammatory cytokines *IL6*, *IL8*, *IL1B* and *TNFA*. All were up-regulated by 3-12-fold in *ATM* KO versus WT cells, whereas the mRNA levels of anti-inflammatory *IL4* were unaffected by ATM status (Figure 1C). The microglial activation phenotypes were rescued upon re-expression of ATM from a doxycycline-inducible piggyBac-transgene (28), showing that the effects were specific for ATM loss (Figure 1A-C; Supplementary Figure S2B).

One indicator of microglial activity is their ability to engulf cell debris, apoptotic and stressed cells, which expose the ‘eat-me’ signal, phosphatidylserine, on their surface (6). Phagocytic properties of WT and *ATM* KO microglia were investigated using 5 μm carboxylated latex beads, which mimic the size of neuronal soma with externalized phosphatidylserine, as substrates (Supplementary Figures S3A and C). Both the percentage of phagocytic cells, further referred to as levels of phagocytosis, and phagocytic activity, which reflects the relative fluorescence intensity of engulfed beads per cell, were measured using flow cytometry (Supplementary Figure S3B). We observed an increase in: (i) the percentage of phagocytic *ATM* KO cells compared to WT, over a 24 h time period, using immunofluorescence and flow cytometry (Figures 1D and 1E) and (ii) the fraction of highly phagocytic *ATM* KO cells, which engulfed more than 2 beads per cell, at different time points compared with WT (Figure 1F). The kinetics of phagocytic changes was linear in both cell lines over a 24 h period. However, to exclude any early apoptotic changes due to uptake of non-digestible substrates, the assays were carried out for 6 h. Also, given that the changes in phagocytic activities were modest, possibly due to the relatively large size of the substrates, levels of phagocytosis were used as the primary readout in subsequent assays. Upon re-expression of ATM, enhanced phagocytic properties of *ATM* KO cells were rescued (Figure 1G; Supplementary Figures S3C and S3D). Similar to *ATM* KO microglia, increased levels of phagocytosis were observed in HMC3 and C20 microglia, in which ATM was knocked down using siRNA (Supplementary Figure S3E-H). These data indicate that ATM-deficient microglia are more efficient phagocytes than their WT counterparts, and this effect is specific to ATM loss and cell line independent.

To investigate whether enhanced phagocytic properties of ATM-deficient microglia are mediated via the loss of ATM protein or its kinase activity, WT cells were treated with a reversible ATM inhibitor, AZD1390 (47), for up to 9 days (Supplementary Figures S4A and B). To rescue the effects of ATM inhibition, AZD1390 was washed out and the cells were allowed to recover for 2 days (Supplementary Figures S4A and B, Release). The levels of ATM autophosphorylation and CHK2 phosphorylation following treatment with camptothecin indicated that kinase inhibition and inhibitor wash-out were successful (Supplementary Figure S4A). We then determined phagocytosis levels of AZD1390-treated WT cells and respective DMSO-treated controls. Although no significant changes in phagocytosis levels were observed at day 1 of inhibitor treatment, we found a 2–2.5-fold increase at days 4-6 in the ATM-inhibited cells compared to the control. Importantly, inhibitor wash-out rescued phagocytosis to the levels of DMSO-treated microglia (Supplementary Figure S4B).

Together, these data indicate that loss of ATM results in persistent microglial activation, which is characterized by increased expression of pro-inflammatory cytokines and enhanced ability to engulf synthetic substrates. These phenotypes are specific to ATM although they may be driven by long-term consequences of ATM loss, such as persistent DNA damage and/or its consequences.

### The non-canonical RELB/p52 NF-κB pathway is activated in ATM-deficient microglia

The NF-κB proteins are central mediators of inflammation in response to tissue damage and infection (48). If mis-regulated, normally protective NF-κB-mediated pro-inflammatory responses can amplify acute or chronic tissue damage, thus driving autoinflammatory and autoimmune disease (34). NF-κB family members include RELA (p65), RELB, c-REL, p50 and p52. The canonical NF-κB pathway provides a rapid and transient response to cytokines, mitogens, and growth factors. These stimuli activate the IκB kinase (IKK) complex, which phosphorylates IκBα, inducing its proteasomal degradation. Loss of inhibitory binding of IκBα to NF-κB proteins results in nuclear translocation of the canonical NF-κB dimers, the most abundant of which are p65-p50 and c-REL-p50. The precursor protein p105 also acts in the NF-κB-inhibitory manner and becomes active upon proteolytic processing into p50 upon stimulation (34).

**Figure 2.**
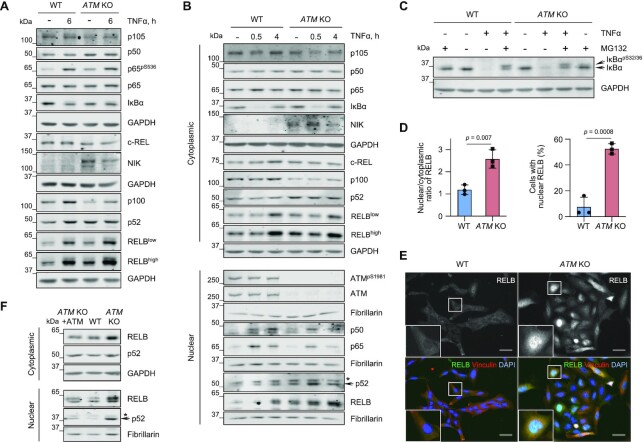
The RELB/p52 non-canonical NF-κB pathway is activated in ATM-deficient microglia. (**A**) Representative immunoblot analysis of NF-κB protein levels in WT and *ATM* KO HMC3 microglia using the indicated antibodies. Positive control: 10 μg/ml TNF-α for 6 h. Loading control: GAPDH. (**B**) Representative immunoblot analysis of nuclear relocalization of NF-κB family members in WT and *ATM* KO HMC3 as in. Positive controls: 10 μg/mL TNF-α for 30 min and 4 h. Loading controls: GAPDH (cytoplasmic), fibrillarin (nuclear). *Non-specific band. (**C**) Representative immunoblot analysis of TNFα-induced proteasomal degradation of IκBα in WT and *ATM* KO HMC3 microglia pre-treated in the presence or absence of proteasomal inhibitor MG-132 (1 μM for 1 h). Positive control: 10 μg/ml TNF-α for 30 min. Loading control: GAPDH. (**D**) Immunofluorescence-based quantification of RELB localization presented as nuclear/cytoplasmic ratio (relative to WT; left) and percentage of cells with nuclear RELB (right) in WT and *ATM* KO HMC3 cells. Mean ± S.D. shown (*n* = 3). Unpaired *t-*test used. (**E**) Representative images of (D). Images in a single Z-plane shown. Scale bar: 50 μm. RELB (green), vinculin (red), DNA (blue). (**F**) Representative immunoblot analysis of RELB levels and localization in cytoplasmic and nuclear extracts of WT, *ATM* KO and *ATM* KO HMC3, in which ATM was re-expressed as in Figure 1A. Loading controls: GAPDH (cytoplasmic), fibrillarin (nuclear). *Non-specific band.

Microglial activation and neuroinflammation are linked to the activities of the p65-p50 heterodimer (49). In addition, p65 is localized to the nucleus in rodent models of A–T (11,12). We therefore investigated whether the canonical NF-κB pathway is activated in ATM-deficient microglia. We first tested whether canonical NF-κB signalling can be induced in WT and *ATM* KO cells upon treatment with the tumour necrosis factor TNFα, a well-established activator of the pathway (34). We observed TNFα-dependent: (i) phosphorylation of p65 at S536 indicative of increased IKK activity (50) and relocalization of p65 in the nucleus (Figure 2A and B; Supplementary Figure S5A–D), (ii) processing of p105 to p50 as evidenced by an increase in p50/p105 ratio and nuclear translocation of p50 (Figure 2A and B; Supplementary Figures S5A and B) and (iii) degradation of the NF-κB-inhibitory protein IκBα that was rescued upon treatment with the proteasomal inhibitor MG-132 (Figures 2B and 2C; Supplementary Figure 5E) in both WT and *ATM* KO microglia. Although we did not observe relocalization of c-REL following TNFα treatment (Figure 2B), its nuclear presence was detected upon treatment of HMC3 microglia with a cocktail of anti-inflammatory cytokines, indicating its context-dependent functions (51). These data indicate that WT and *ATM* KO HMC3 microglia are proficient in TNFα-induced canonical NF-κB signalling.

Next, we determined whether the canonical NF-κB pathway is activated in basal conditions in the absence of ATM. While we observed a modest increase in p65 phosphorylation at S536 and the processing of p105 into p50 in *ATM* KO cells as compared to WT, no degradation of inhibitory IκBα was seen. Consequently, we did not detect any basal relocalization of p65, c-REL and p50 into the nucleus (Figure 2A-C; Supplementary Figure S5A-E). A similar lack of basal nuclear translocation of p65 was seen in *ATM* KO C20 microglia, confirming that the effect was not cell-line specific (Supplementary Figure S5F-H). Together, these data indicate that no basal activation of the canonical NF-κB pathway occurs in the absence of ATM.

Non-canonical NF-κB signalling is activated downstream of cell surface receptors of the tumour necrosis factor receptor (TNFR) superfamily, such as BAFFR, CD40, LTβR, and RANK. Pathway activation is dependent on stabilization of the NIK (NF-κB-inducing) kinase, which phosphorylates IKKα, promoting IKKα-mediated phosphorylation of p100 and its cleavage-dependent processing to p52. RELB-p52 is the major non-canonical heterodimer that drives transcriptional programmes (35).

To investigate whether the non-canonical NF-κB pathway is activated in ATM-deficient microglia under basal conditions, we first determined the levels of NIK and the efficiency of p100 processing by immunoblotting. NIK protein levels were increased in the *ATM* KO cells, compared to WT, indicating its enhanced stability, although additional modes of regulation, for example at the level of protein expression, cannot be excluded (Figure 2A and B). In addition, a reduction in basal levels of p100 and a concurrent increase in p52 levels were observed in *ATM* KO microglia compared to the WT control, indicating enhanced p100 processing (Figure 2A and B; Supplementary Figure S5A and B). We also determined the basal levels of RELB and its cellular localization in WT and *ATM* KO HMC3 cells (Figure 2A, B, D–F; Supplementary Figure S6A and B). The specificity of the antibody against RELB, which also detects various post-translationally modified forms of the protein (52), was confirmed using siRNA-mediated knockdown (Supplementary Figure S6A). In agreement with the *de novo* synthesis of RELB prior to nuclear translocation (35), basal RELB levels were increased in the absence of ATM compared to the WT control (Figure 2A and B; Supplementary Figure S6A). Additionally, RELB was detected in the nucleus of *ATM* KO cells whereas almost no nuclear RELB was found in the WT control (Figure 2B; Supplementary Figure S6A). The findings on basal nuclear relocalization of RELB in the absence of ATM were independently confirmed using immunofluorescence. An increase in the nuclear to cytoplasmic (N/C) ratio and in the percentage of cells with nuclear RELB was observed in *ATM* KO cells versus the WT control (Figure 2D and E). Noteworthy, the increased levels and nuclear translocation of RELB and p52 observed in ATM *KO* cells were rescued upon re-expression of ATM (Figure 2F; Supplementary Figure S6B). Finally, a similar basal increase in nuclear RELB was seen in *ATM* KO C20 microglia (Supplementary Figure S6C and D).

To confirm and extend these results, RELB levels and localization were determined in WT cells treated with the ATM kinase inhibitor, AZD1390, for up to 9 days (Supplementary Figure S4A). A gradual increase in RELB expression and nuclear translocation was observed at days 6-9 of treatment, whereas no changes were seen in the DMSO-treated control (Supplementary Figure S6E and F). Together, these results show that loss of ATM function results in specific chronic activation of the RELB/p52 non-canonical NF-κB pathway.

### The non-canonical NF-κB pathway controls microglial activation in the absence of ATM

**Figure 3.**
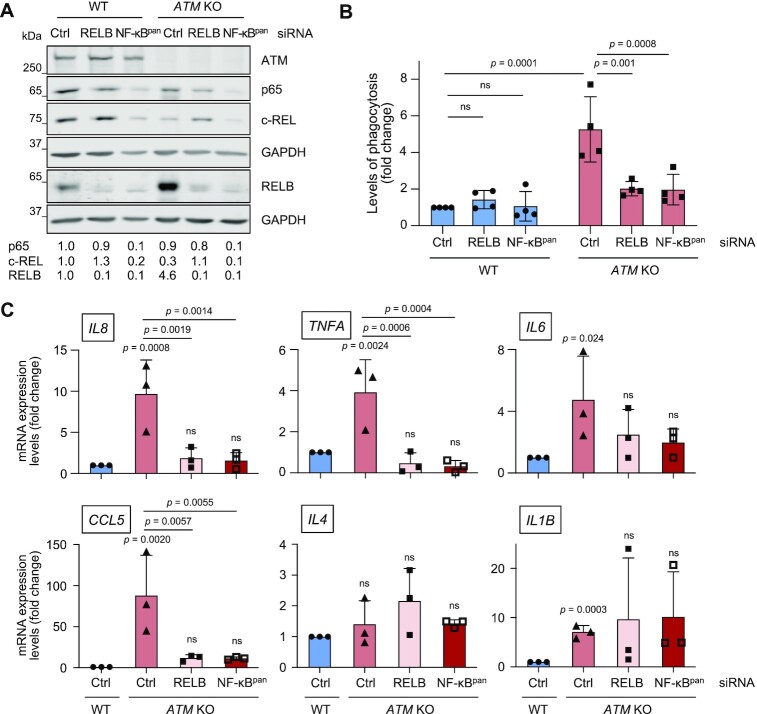
Microglial activation in the absence of ATM is mediated via RELB. (**A**) Representative immunoblot analysis of siRNA-mediated silencing of RELB or all NF-κB members (NF-κB^pan^) in WT and *ATM* KO HMC3 cells. Loading control: GAPDH. Quantification of protein levels is relative to loading control. (**B**) Phagocytosis levels (5 μm beads) of WT and *ATM* KO HMC3 cells treated with control (Ctrl), RELB or NF-κB^pan^ siRNA. Phagocytosis is relative to WT HMC3 treated with Ctrl siRNA. Mean ± S.D. shown (*n* = 4). Two-way ANOVA with Tukey's multiple comparison's test used. (**C**) RT-qPCR analysis of the indicated cytokines as in (B). Expression is relative to WT HMC3 treated with control siRNA (Ctrl). Reference gene: *RSP13*. Mean ± S.D. shown (*n* = 3). Two-way ANOVA with Tukey's multiple comparison's test used.

Having observed the stabilization of NIK kinase and nuclear translocation of RELB in the absence of ATM, we aimed to determine whether RELB-containing complexes might be responsible for microglial activation. Therefore, the phagocytic properties and expression levels of several pro- and anti-inflammatory cytokines were investigated in WT and *ATM* KO microglia following siRNA-mediated knockdown of RELB. In addition, siRNA that targets all five NF-κB subunits (NF-κB^pan^) was used to probe for synergistic effects of the canonical and non-canonical pathways (Figure 3A–C). The knockdown of RELB significantly reduced phagocytosis levels in *ATM* KO cells to levels typical of WT cells. No effect of RELB loss on phagocytic properties of WT cells was observed, indicating that the phagocytic properties of ATM-deficient microglia are modulated via RELB (Figure 3B).

Next, we studied the effect of RELB depletion on cytokine expression. As expected, siRNA transfection moderately enhanced the fold change difference in mRNA levels of pro-inflammatory cytokines *IL6*, *IL8*, *TNFA, CCL5* and *IL1B* but not anti-inflammatory *IL4* in *ATM* KO versus WT control, as compared to that seen in unchallenged cells (Figure 3C, compare Ctrl siRNA-treated *ATM* KO versus WT cells with the results in Figure 1C). Importantly, upon RELB knockdown, the high expression levels of NF-κB-dependent cytokines *IL8* and *TNFA* in Ctrl-siRNA treated *ATM* KO microglia was reduced to similar levels as observed in Ctrl-siRNA treated WT cells. Unexpectedly, only a weak dependence of *IL6* expression on RELB status in *ATM* KO cells was observed, and this may be due to the existence of multiple autocrine positive feedback loops, including that regulated by STAT3 (53). Expression of the chemokine *CCL5* was reduced in the absence of ATM and RELB but to a lesser extent, in agreement with it being cooperatively regulated by NF-κB and interferon-regulatory factors IRF1, IRF3 and IRF7 (54). In contrast, mRNA expression levels of *IL1B* that is negatively regulated by NF-κB upon its chronic activation (55), and anti-inflammatory *IL-4* (56), remained unchanged in *ATM* KO cells treated with RELB siRNA (Figure 3C). One important observation is that both phagocytic properties and expression of pro-inflammatory cytokines were reduced at similar levels following treatment of ATM-deficient microglia with siRNA against RELB and all NF-κB subunits, confirming the dependency of these phenotypes on RELB-containing complexes (Figure 3B and C).

These data demonstrate that the RELB/p52 non-canonical NF-κB pathway promotes microglial activation in the form of enhanced phagocytic clearance and expression of pro-inflammatory cytokines in the absence of ATM. We therefore further investigated the mechanisms of RELB/p52 activation in ATM-deficient microglia.

### RELB/p52-dependent activation of ATM-deficient microglia is mediated by NIK kinase

**Figure 4.**
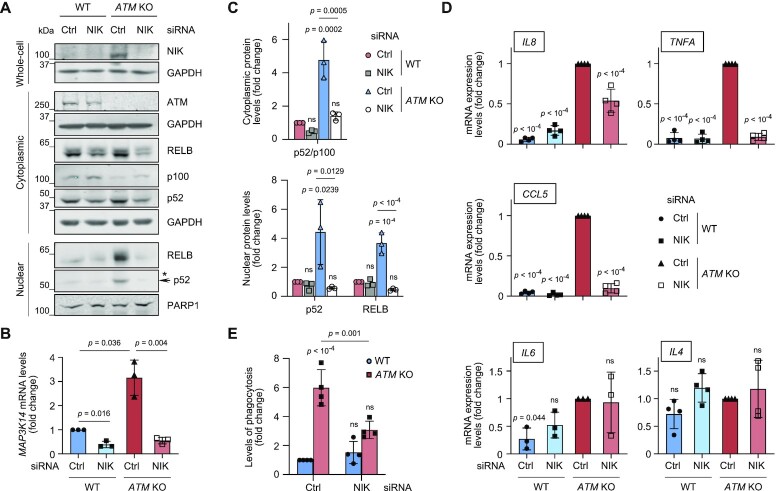
Microglial activation and non-canonical NF-κB signalling in ATM-deficient cells are mediated by NIK kinase. (**A**) Representative immunoblot analysis of the non-canonical NF-κB pathway proteins in whole-cell, cytoplasmic and nuclear extracts of NIK-deficient WT and *ATM* KO HMC3 microglia. Loading controls: GAPDH (whole-cell, cytoplasmic), PARP1 (nuclear). *Non-specific band. (**B**) RT-qPCR analysis of NIK knockdown efficiency in WT and *ATM* KO HMC3 microglia as in (A). Expression is relative to Ctrl siRNA-treated WT HMC3. Reference gene: *RSP13*. Mean ± S.D. shown (*n* = 3). Two-way ANOVA with Tukey's multiple comparison's test was used for 2^-ΔCt^ values. **C** Quantification of protein levels as in (A). Data are relative to loading control. Mean ± S.D. shown (*n* = 3). Two-way ANOVA with Tukey's multiple comparison's test used (*P-*values are shown relative to WT Ctrl siRNA). (**D**) RT-qPCR analysis of the indicated cytokines as in (A). Expression is relative to Ctrl siRNA-treated *ATM* KO HMC3. Reference gene: *RSP13*. Mean ± S.D. shown (*n* = 4 except for *IL6* where *n* = 3). Two-way ANOVA with Tukey's multiple comparison's test used. (**E**) Phagocytosis levels (5 μm beads) of cells treated as in (A). Phagocytosis is relative to Ctrl siRNA-treated WT HMC3, in which 3.5 ± 1.7% of cells are phagocytic. Mean ± S.D. shown (*n* = 4). Two-way ANOVA with Tukey's multiple comparison's test used.

To determine whether enhanced RELB/p52 signalling in the absence of ATM is dependent on NIK kinase, we used siRNA to knock down NIK and investigated the microglial activation phenotypes (Figure 4A–E). As expected from the dependence of processing of p100 to p52 on the NIK-IKKα axis (35), NIK downregulation resulted in the modest accumulation of p100 and consequent reduction in p52 protein levels in the cytoplasm of both WT and *ATM* KO cells (Figure 4A–C). Importantly, NIK loss rescued: (i) the nuclear translocation of both p52 and RELB (Figure 4A and C), (ii) the expression of NF-κB-dependent pro-inflammatory cytokines *IL8, TNFA* and *CCL5*, whereas there was no effect on the levels of *IL6* that was not regulated in RELB-dependent manner (Figure 3C) and anti-inflammatory *IL4* (Figure 4D) and (iii) the levels of phagocytosis (Figure 4E) in *ATM KO*, and not WT microglia. These data show that RELB/p52 non-canonical NF-κB signalling and microglial activation in the absence of ATM are dependent on NIK kinase and are likely to be regulated, at least in part, via extracellular signalling cues.

### Persistent DNA damage promotes RELB/p52 NF-κB signalling and microglial activation

The continuous occurrence of DNA damage requires proficient DNA damage signalling and repair, including ATM-dependent responses, to maintain genome stability. A–T cells are therefore characterized by enhanced oxidative stress and delayed repair of a subset of specialized DNA lesions (2,3,57). Importantly, DNA damage is thought to activate the non-canonical NF-κB pathway, although the mechanism of this activation is unclear (48). We therefore set out to investigate whether DNA damage might drive RELB/p52-dependent activation of microglia.

**Figure 5.**
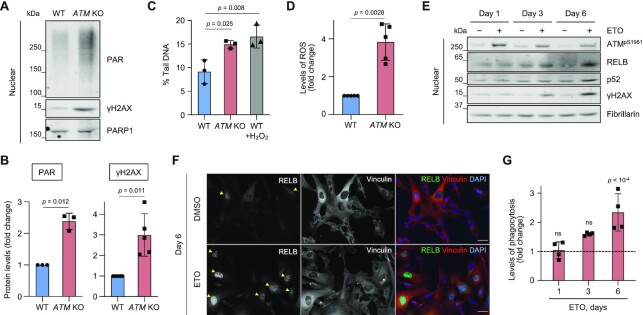
Persistent DNA damage promotes microglial activation and non-canonical NF-κB signalling. (**A**) Representative immunoblot analysis of DNA damage response markers in nuclear extracts of WT and *ATM* KO HMC3 cells using the indicated antibodies. Loading control: PARP1. (**B**) Quantification of protein levels as in (A). Data are relative to loading control. Mean ± S.D. shown (*n* = 3 for PAR and *n* = 5 for γH2AX). One sample *t-*test used. (**C**) Levels of endogenous DNA damage in WT and *ATM* KO HMC3 detected by alkaline Comet assays and presented as % tail DNA. Positive control: 2 μM H_2_O_2_ for 5 min. Mean ± S.D. shown (technical duplicates per experiment, *n* = 3). One-way ANOVA with Dunnett's multiple comparison's test used. (**D**) Levels of intracellular reactive oxygen species (ROS) in WT and *ATM* KO HMC3 measured using DCF-DA probe and flow cytometry. ROS levels are relative to WT HMC3. Mean ± S.D. shown (*n* = 5). One sample *t-*test used. (**E**) Representative immunoblot analysis of DNA damage response markers and RELB/p52 levels in nuclear fractions of WT HMC3 microglia treated with 0.5 μM etoposide (ETO) for up to 6 days. Control: DMSO. Loading control: fibrillarin. (**F**) Representative images of cells treated as in (E). Images in a single Z-plane shown. Arrowheads indicate cells with nuclear RELB. Scale bar: 50 μm. RELB (green), vinculin (red), DNA (blue). (**G**) Phagocytosis levels (5 μm beads) of WT microglia treated as in (E). Phagocytosis is relative to DMSO control at respective time points (dashed line). Mean ± S.D. shown (*n* = 3). One-way ANOVA with Tukey's multiple comparison's test used.

To determine whether *ATM* KO microglia possess increased levels of spontaneous DNA damage, we analysed the cells for several DNA damage markers. Protein poly[ADP-ribosyl]ation (PAR) that occurs at sites of DNA single- (SSBs) and double-strand breaks (DSBs) and phosphorylation of histone variant H2AX at S139 (γH2AX), which marks DSBs (4,58), were increased in ATM-deficient cells (Figures 5A and 5B). Additionally, the basal levels of DNA damage were directly measured using alkaline single-cell gel electrophoresis, which detects alkali labile SSBs and DSBs (59). Basal levels of DNA damage were elevated in *ATM* KO microglia compared to the WT control (Figure 5C). We also analysed the levels of intracellular reactive oxygen species (ROS), which serve as one of the sources of DNA damage in ATM-deficient cells. Increased intracellular ROS were detected in *ATM* KO microglia, in comparison with WT (Figure 5D).

To induce persistent low-level DNA damage, as seen in ATM-deficient microglia, WT HMC3 cells were treated with a low dose of etoposide for 6 days, a topoisomerase II inhibitor that induces DSBs (60) (Figure 5E–G; Supplementary Figure S7A–D). The presence of DNA damage was confirmed by the increased levels of γH2AX and autophosphorylation of ATM at S1981 (Figure 5E; Supplementary Figures S7A and S7B). The concentration of etoposide was sufficiently low to ensure that no visible apoptosis and/or necrosis was observed following 6 days of continuous treatment, as adjudged by the lack of cleaved caspase-3 positive staining or condensed and fragmented nuclei (Supplementary Figure S7A). Importantly, the treatment resulted in a time-dependent increase in: (i) the levels of nuclear RELB and p52 in the absence of nuclear translocation of p65 (Figures 5E and 5F; Supplementary Figure S7B–D) and (ii) the levels of phagocytosis as compared to respective DMSO controls (Figure 5G). The kinetics of DNA damage-induced changes in nuclear translocation of RELB and phagocytosis was similar to that observed in response to treatment with AZD1390 (Supplementary Figures S4A, S4B, S6E, and S6F).

To determine whether persistent oxidative stress could induce microglial activation, WT HMC3 microglia were treated with *tert*-butyl hydroperoxide for up to 6 days (tBHP; Supplementary Figure S7E–H). A concentration-dependent increase in the levels of ROS upon treatment with tBHP was detected, which was comparable to that seen in *ATM* KO versus WT microglia (compare Supplementary Figure S7G, 250 μM tBHP with Fig. 5D). However, we did not observe significant changes in the levels of: (i) DNA damage response markers, including PAR and γH2AX following tBHP treatments (Supplementary Figure S7E and F), or (ii) phagocytosis (Supplementary Figure S7H). These results indicate that oxidative damage generated in our experiments is within the cellular capacity of cells to repair it, and it does not serve as a source of measurable SSBs and/or DSBs. More importantly, microglial activation in *ATM* KO cells was not due to increased ROS levels *per se*. Indeed, no changes in phagocytosis levels of *ATM* KO microglia was detected upon reducing intracellular ROS using a superoxide radical scavenger, Tiron (61) (Supplementary Figure S7I). We therefore propose that persistent low-level DNA damage, and/or its consequences, associated with ATM loss underpin microglial activation mediated by the non-canonical RELB/p52 pathway.

### ATM-deficient microglia excessively engulf neurites

In addition to secretion of pro-inflammatory cytokines and oxidative species, chronically activated microglia can contribute to neurodegenerative pathologies by: i) defective phagocytosis of dead and dying cells that leads to uncontrolled release of neurotoxic compounds and ii) excessive phagocytosis of neuronal synapses, axons and dendrites (62–65). To investigate the effects of microglial ATM deficiency on neuronal engulfment, we employed post-mitotic dopaminergic neurons obtained by differentiation of human mesencephalic LUHMES progenitors (66) (Supplementary Figure S8A and B). To model ATM’s loss-of-function, both inhibition of kinase activity and gene knockout approaches were used. To provide a model in which ATM kinase activity was inhibited, post-mitotic neurons were maintained in the presence of AZD1390 during differentiation days 2–5. After AZD1390 was washed out, ATM activity remained inhibited for at least 48 h, during which time the ATM-inhibited neurons were used in assays (Supplementary Figure S8C). The 3-day inhibition of ATM did not induce measurable neuronal apoptosis in post-mitotic LUHMES (Supplementary Figure S8D). A similar lack of apoptosis/necrosis was seen in two clonal *ATM* KO LUHMES cell lines that were generated using CRISPR/Cas9 (Supplementary Figure S8E and F). In addition, we observed no damaging effect of factors secreted by WT or *ATM* KO microglia on ATM-deficient neurons using a transwell assay (Supplementary Figures S8G and S8H).

We observed that loss of ATM in microglia results in morphological changes associated with their activation (Supplementary Figure S9A and B). Such changes include microglial transition to a flat, round morphology in response to pro-inflammatory stimuli, and should be studied in co-cultures of microglia with neurons and/or astrocytes or *in vivo* (67,68). The results of morphological analyses revealed a trend towards an increase in circularity in *ATM* KO HMC3 microglia, consistent with a more amoeboid morphology that is indicative of microglial activation (Supplementary Figures S9A and S9B).

**Figure 6.**
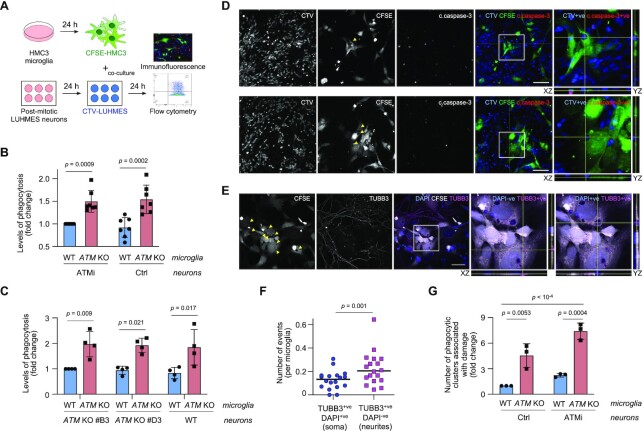
Neurotoxicity of ATM-deficient microglia is linked with aberrant phagocytosis of healthy neuronal soma and neurites. (**A**) Schematic of neuronal-microglial co-culture assays. Phagocytic uptake of LUHMES neurons by HMC3 microglia was analysed by flow cytometry and immunofluorescence. (**B**) Phagocytosis levels (CTV-stained post-mitotic LUHMES) of WT and *ATM* KO CFSE-labelled HMC3 cells using post-mitotic LUHMES neurons. LUHMES cells were pre-treated with AZD1390 for 3 days and released from inhibition prior to setting up the co-cultures (ATMi) or left untreated (Ctrl). Phagocytosis is relative to WT HMC3/ATMi LUHMES. Mean ± S.D. shown (*n* = 4). Two-way ANOVA with Sidak's multiple comparisons used. (**C**) Phagocytosis levels (CTV-stained post-mitotic LUHMES) of WT and *ATM* KO CFSE-labelled HMC3 cells using WT and two independent clones of *ATM*KO post-mitotic LUHMES neurons as substrates. Phagocytosis is relative to WT HMC3/*ATM* KO LUHMES (clone #B3), in which 9.9 ± 3.4% of cells are phagocytic. Mean ± S.D. shown (*n* = 4). Two-way ANOVA with Sidak's multiple comparisons used. (D, E) Representative immunofluorescence images of phagocytosis events by *ATM* KO microglia as in (B). Confocal Z-stack compression images and relevant XY and YZ projections shown. Scale bar: 50 μm. (**D**) Arrows indicate microglial phagolysosomes containing engulfed neuronal bodies (CTV+ve), which are positive (top) or negative for cleaved caspase-3 (bottom). CFSE-labelled *ATM* KO HMC3 microglia (CFSE; green), cleaved caspase-3 (c.caspase-3; red), CTV-labelled LUHMES neurons (CTV; blue). (**E**) Arrows indicate microglial phagolysosomes containing neuronal soma (TUBB3+ve, DAPI+ve) and neurites (TUBB3+ve, DAPI-ve). CFSE-labelled *ATM* KO HMC3 microglia (CFSE; green), β3-tubulin (TUBB3; red), DAPI (blue). (**F**) Quantification of phagocytic events as in (E). Distribution of TUBB3+ve DAPI+ve versus TUBB3+ve DAPI-ve events per microglia shown (*n* = 20 fields of view from two independent biological experiments). Wilcoxon matched-pairs signed rank test used. (**G**) Quantification of phagocytic microglial clusters associated with damage to the neuronal network as in (B). Data are relative to WT microglia co-cultured with untreated LUHMES post-mitotic neurons (Ctrl). Mean ± S.D. shown (*n* = 3). Two-way ANOVA with Tukey's multiple comparison's test used.

For the phagocytosis assays, microglia and neurons were labelled with the fluorescent live dyes carboxyfluorescein succinimidyl ester (CFSE) and CellTrace Violet (CTV), respectively, and cultured together for 24 h (Figure 6A). The presence of CTV-positive neuronal material within CFSE-positive microglia (double positive cells) was assessed by flow cytometry. ATM *KO* microglia consistently engulfed (50 ± 13)% more ATM-inhibited neuronal material than WT microglia (Figure 6B, left). Unexpectedly, a similar increase in the engulfment of healthy WT neurons by *ATM* KO microglia, as compared with WT microglia, was observed (Figure 6B, right). Similarly, we detected a 1.9–2.0-fold increase in the phagocytic uptake of both WT and *ATM* KO neuronal material by *ATM* KO, in comparison with WT, microglia (Figure 6C). These results indicate that microglial phagocytosis in the absence of ATM is regulated via cell-intrinsic mechanisms.

To investigate the types of phagocytic events that occurred in co-cultures of WT or *ATM* KO microglia with WT or ATM-deficient neurons we used confocal microscopy. ATM-deficient microglia were found to uptake both apoptotic (positive for cleaved caspase-3) and viable (negative for cleaved caspase-3 and non-pyknotic) neuronal soma, although both types of events were relatively rare (Figure 6D). To determine whether some of the material readily phagocytosed by the *ATM* KO microglia included neurites, neuronal networks were visualized using an antibody against β3-tubulin following co-culture with CFSE-labelled microglia (Figure 6E). We observed that the *ATM* KO microglia demonstrated a significantly increased uptake of neurites (DAPI negative, β3-tubulin positive) compared to neuronal soma (DAPI positive, β3-tubulin positive) (Figure 6F). In addition, microglial clusters that formed specifically in co-cultures with neurons were detected (Supplementary Figure S9C). The number of microglial clusters that contained phagolysosomes showed a tendency to increase in co-cultures of both WT and ATM-deficient neurons with *ATM* KO, but not WT, microglia (Supplementary Figure S9D). Importantly, clusters of *ATM* KO microglia, compared with WT microglia, were associated with disruptions in the neuronal network and damaged neurites when cultured with ATM-deficient neurons. Similar effects, albeit to a lesser extent, were observed in the case of healthy neurons (Figure 6E and G). Taken together, these data demonstrate the intrinsic capacity of activated *ATM* KO microglia to aberrantly engulf neuronal soma and neurites *in vitro*.

### Non-canonical NF-κB pathway is activated in cerebellar microglia of individuals with A–T

**Figure 7.**
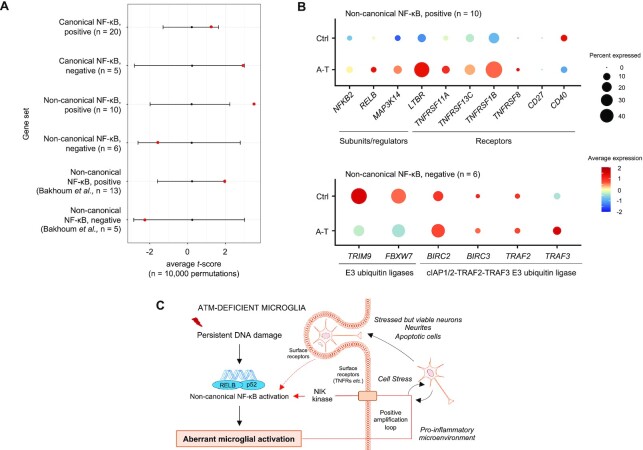
Non-canonical NF-κB signalling is upregulated in cerebellar microglia of individuals with A–T. (**A**) Average *t-*scores for canonical and non-canonical NF-κB gene sets from differential gene expression analyses of A–T versus control (Ctrl) cerebellar microglia. Black bars represent the null distribution of gene set average *t-*scores (mean ± 2SD). Red points represent the observed gene set average *t-*scores. Positive/negative average *t*-scores are indicative of increased/decreased fold change in gene expression in A–T versus Ctrl. The number of genes in a dataset is indicated as n. For *P-*values see Supplementary Table S3. (**B**) Dotplots showing average expression and percent cells expressed for individual genes involved in activation (positive) and inhibition (negative) of the non-canonical NF-κB pathway in A–T versus Ctrl cerebellar microglia. Average expression is represented by the *z-*score calculated by centering and scaling across microglia from A–T and Ctrl in cerebellum and prefrontal cortex. (**C**) Proposed mechanism for the aberrant activation of microglia in the absence of ATM kinase. Persistent DNA damage and/or its consequences associated with the loss of ATM, or its kinase activity, results in activation of the RELB/p52 non-canonical NF-κB pathway. This leads to microglial activation characterized by increased expression of pro-inflammatory cytokines and enhanced phagocytic clearance properties. The release of interleukins, chemokines, and reactive oxidative species in the extracellular compartment could promote and amplify sustained non-canonical NF-κB activation via, for example, TNFR-family CD40, LTβR or TNFR2 receptors and via NIK kinase-dependent signalling (arrows in red). Cell-intrinsic effects of ATM loss together with chronic inflammation could lead to aberrant phagocytic uptake of stressed but viable neurons and neurites. The engulfment of neuronal material could provide a secondary positive feedback loop to the non-canonical NF-κB signalling, thus contributing to progressive neuronal damage in A–T. Positive feedback loops are shown in red arrows. Dashed arrows indicate unestablished connections. The image was created using Motifolio illustration toolkits.

To determine whether the NF-κB pathway is regulated in human disease, we analysed the microglia transcriptomes from a single-nucleus RNA-sequencing dataset of human cerebellum (CB) from individuals with A–T and healthy controls (33). Enrichment analyses of canonical and non-canonical NF-κB pathway gene sets of A–T versus control cerebellar microglia were performed. No enrichment of the canonical NF-κB pathway was detected, and no increase in the expression of: (i) genes directly involved in this pathway, such as *RELA*, *IKBKB*, *IKBKG* and *MAP3K7* (encoding p65, IKK-β, IKK-γ/NEMO and TAK1, respectively) and (ii) *NFKBIA* (encodes for IkBα), which is a target of p65 (69,70), was observed in A–T versus control (Figure 7A; Supplementary Figure S10A; Supplementary Tables S2–S4). With regard to the non-canonical NF-κB pathway, gene set enrichment analyses showed that the positive regulation of the pathway was significantly increased in A–T cerebellar microglia compared to controls (Bonferroni adjusted *P* = 0.027, one-sided *t-*score permutation test; Figure 7A; Supplementary Tables S2 and S3). Expression of most of the genes in the pathway, including non-canonical NF-κB subunits, regulators and stimulating receptors, showed a tendency for upregulation in A–T cerebellar microglia (Figure 7B; Supplementary Table S4). Among the negative regulators of RELB/p52 NF-κB signalling, the expression of *FBXW7* and *TRIM9*, which encode E3 ubiquitin ligases that promote proteasomal degradation of p100 (71) and inhibit ubiquitin-mediated proteolysis of p100 into p52 (72,73), respectively, was significantly reduced in A–T (Figure 7B; Supplementary Table S4).

To extend these findings, we analysed the enrichment and expression levels of non-canonical NF-κB target genes previously identified in chromosomally unstable metastatic breast cancer cells (36). Several RELB target genes that were positively regulated or suppressed in breast cancer cells showed a tendency to be up- and down-regulated, respectively, in A–T versus control cerebellar microglia (Supplementary Figure 10B; Supplementary Table S4).

In summary, from these data we suggest that non-canonical NF-κB signalling is activated in cerebellar microglia from individuals with A–T. Aberrant upregulation of the non-canonical NF-κB pathway linked with microglial dysfunction may therefore contribute to cerebellar degeneration in A–T.

## DISCUSSION

A–T is a prototypical genomic instability disorder characterized by progressive cerebellar neurodegeneration. Neuroinflammation associated with genomic instability is increasingly recognized as a possible driver of progressive neurodegeneration (74). Previously, activation of microglia was observed in rodent models of A–T (10–13). In addition, morphological observations indicative of microglial activation were made in post-mortem brain samples from individuals with A–T (75). Here, using immortalized human cell models, we show that ATM-deficient microglia exhibit multiple features of activation, including increased expression of pro-inflammatory cytokines, cell surface CD40 and lysosomal CD68 markers and increased phagocytic clearance.

In the context of increased oxidative stress and DNA damage associated with ATM deficiency, the NF-κB proteins represent the fundamental mediators of inflammation. Although the canonical NF-κB pathway was inducible in both WT and *ATM* KO cells in response to TNFα, we did not observe degradation of IκBα or the nuclear translocation of the canonical NF-κB subunits in the absence of ATM under basal conditions. Despite that NF-κB responses to proinflammatory stimuli and genotoxic stress are known to be functionally distinct, proteasomal degradation of IκBα is necessary for p65 NF-κB activation in both contexts (76). In contrast, stabilization of the NIK kinase, nuclear localization of RELB, and proteolytic processing of p100 to p52 were apparent in ATM-deficient microglia, confirming basal activation of the non-canonical NF-κB pathway. Microglial activation in the absence of ATM was dependent on RELB and mediated by the NIK kinase. Our findings using microglial cell lines were strengthened by the analysis of single-nuclear RNA-seq data from brain tissues of individuals with Ataxia-telangiectasia. These results are suggestive of the activation of non-canonical NF-κB signalling in A–T cerebellar microglia.

At the present time, the role of non-canonical NF-κB signalling in microglia is poorly understood. However, the choice of RELB-dependent versus p65-mediated NF-κB pathway in the absence of ATM is clearly defined by the specificity of the upstream signal. We find that in ATM-proficient microglia, the RELB/p52 signalling was activated following long-term induction of low-level unrepaired DNA damage, and not by ROS. Similar slow (days rather than minutes/hours) kinetics of RELB nuclear relocalization was observed upon ATM inhibition. Interestingly, activation of NF-κB responses to γ-irradiation and topoisomerase poisons was dependent on the formation of DSBs, while oxidative stress and SSBs were not sufficient to induce such responses, and had slow kinetics (77,78). The results lead us to suggest that specific activation of the RELB/p52-mediated non-canonical NF-κB pathway in microglia is linked with persistent DNA damage associated with loss of ATM function (Figure 7C). The detailed mechanistic understanding of the primary events and the signalling cascade that trigger this activation are presently unclear. One possibility is that aberrantly localized cytosolic DNA, which arises from micronuclei and damaged mitochondria in A–T (10,12,14,16,17), stimulates the non-canonical NF-κB pathway in human microglia. This stimulation may occur in a manner similar to that observed in ATM inhibited murine microglia, in which canonical NF-κB activation was mediated by STING (12). Indeed, chromosomal instability was shown to drive STING-dependent non-canonical NF-κB responses in tumour cell models, aiding chronic activation of innate immune signalling associated with invasion and metastasis (36). However, mechanistic links between cytosolic DNA signalling and the non-canonical NF-κB pathway are yet to be defined (74). Alternatively, poly[ADP-ribose] polymerase-1, a DNA strand break sensor, which cooperates with ATM to regulate the canonical NF-κB pathway, may play a role in RELB/p52 NF-κB signalling in the absence of ATM (79,80).

Given the chronic nature of pro-inflammatory NF-κB signalling in the absence of ATM, it is important to consider the consequences of such responses. In contrast to inflammation-induced pro-survival function of NF-κB, canonical p65 signalling following prolonged genotoxic exposure leads to anti-apoptotic gene repression and cell death (69,81). Conversely, the non-canonical NF-κB pathway promotes cell survival albeit at the expense of disease-promoting chronic inflammation and autoimmunity (35). NIK- and RELB/p52-mediated pathways supress p65-dependent production of type I interferons and pro-inflammatory cytokines (82,83). In addition, exchange of p65/p50 to RELB/p52 dimers at promoters was observed during prolonged genotoxic exposure and in certain cell types, for example, during maturation of dendritic cells (84,85). It is therefore possible that RELB/p52-mediated signalling in the absence of ATM provides a tolerance mechanism for limiting p65-driven pro-apoptotic responses in response to persistent DNA damage. Indeed, human microglia are highly immune reactive compared to their rodent counterparts (86). The differences in the threshold for execution of pro-survival strategies may explain why our findings of non-canonical NF-κB activation in ATM-deficient human microglia differ from the previously observed activation of p65 in rodent models of A–T (10,12). In addition, inhibition of ATM kinase activity for 48 h in murine microglia may not be fully sufficient for detecting steady-state changes in chronic inflammatory responses (12). Indeed, we find that chronic activation of the non-canonical NF-κB pathway is induced after 6–9 days of ATM kinase activity inhibition, and is apparent under basal conditions in *ATM* KO microglia. Noteworthy, activation of p65 NF-κB signalling should be adjudged by nuclear relocalization/DNA binding of p65 and not by its phosphorylation (17), as the latter is not sufficient for the pathway activation.

Despite co-opting pro-survival strategies, chronic activation of non-canonical NF-κB signalling is nevertheless accompanied by aberrant clearance of neurites, and to a much lesser extent neuronal soma, by *ATM* KO, but not WT, microglia in the context of chronic inflammation. Our results indicate that highly phagocytic clusters of *ATM* KO microglia are associated with, and may drive, the damage of neuronal network via aberrant phagocytosis. Interestingly, clusters of activated microglia were previously detected in the cerebellum of A–T patients, although their function was not determined (75). Importantly, aberrant microglial pruning of neurites and synapses drives neurodegeneration in murine models of progranulin deficiency, Alzheimer's disease and multiple sclerosis, and blocking microglial phagocytosis mitigates the pathological consequences (87–89).

The possibility that clusters of highly phagocytic *ATM* KO microglia contribute to neuronal damage via local secretion of cytokines cannot be excluded. Indeed, in murine model of A–T, microglia-induced neurotoxicity was dependent on secretion of IL-1β (12). In addition, microglia from individuals with A–T show enrichment of genes associated with cytokine production (33). We propose that initial activation of the non-canonical NF-κB pathway in the presence of unrepaired DNA damage could promote the release of cytokines in the extracellular compartment, thus establishing the local pro-inflammatory environment. Such inflammation could lead to cellular stress and cooperate with the aberrant phagocytic activities of microglia (Figure 7C). Our findings that activation of *ATM* KO microglia is rescued by NIK kinase depletion confirm the contribution of cell surface receptor-cytokine interactions to inflammation. These interactions may involve: (i) the TNFR receptor CD40, cell surface expression of which is increased in *ATM* KO HMC3 microglia, or (ii) other non-canonical NF-κB-stimulating receptors, such as LTβR or TNFR2, as mRNA expression of their respective genes was found to be significantly upregulated in cerebellar A–T microglia. The result of this positive feedback loop in A–T microglia would be the amplification of non-canonical NF-κB signaling and chronic neuroinflammation, possibly contributing to disease progression. The phagocytic clearance of neuronal material would further perpetuate the non-canonical NF-κB signalling pathways and inflammation (Figure 7C). Importantly, expression of genes associated with microglial activation, neuroinflammation and phagocytosis was increased in cerebellar microglia from individuals with A–T (33).

The observation that loss of ATM drives normally neuroprotective microglia to become dysfunctional, are in general agreement with the neurotoxicity of Atm-inhibited murine microglia cultured *ex vivo* (12). There are, however, some mechanistic differences. Following ATM inhibition, neurotoxicity is dependent on secretion of IL-1β by microglia in the absence of contact with neurons and is controlled by the STING-p65 canonical NF-κB pathway. In contrast, in human *ATM* KO microglia, we did not observe global damage of ATM-deficient neurons via secretion. One possibility is that the use of an ATM inhibitor, which acts as a dominant negative mutant of ATM (90), exacerbated the toxicity of murine microglia and contributed to neuronal susceptibility to inflammation. In the present work, the levels of phagocytosis of both damaged and live neuronal material by *ATM* KO microglia were 50–100% higher compared to WT cells. These observations are somewhat at odds with the recent data on *Atm^–/^^–^* cerebellar microglia, which are activated, as adjudged by their morphology and increased motility, but show reduced phagocytosis in comparison with controls (13). While we cannot explain these differences, the expression of genes involved in phagocytosis was upregulated in cerebellar microglia from individuals with A–T, indicating enhanced phagocytic processes in this disease (33). Although the contribution of *ATM* KO microglia to neuroinflammation and phagocytosis observed in this work may appear to be modest, we suggest that even small increases in dysfunctional microglial clearance could lead to damage of PC and granule cells in a human brain. These findings are consistent with the slow progressive nature of neurodegeneration observed in individuals with A–T.

Microglia are known to dynamically interact with both the soma of PCs and their dendritic arborizations *in vivo*, and there is increasing evidence that these interactions might be dysfunctional in disease (91). For example, in a mouse model of Niemann Pick Type-C disease, which is associated with genetic defects in lysosomal storage, activated cerebellar microglia showed increased engulfment of PC dendrites, contributing to neuronal degeneration (92). Interestingly, microglia show high regional diversity, with cerebellar microglia being the most distinct based on their gene-expression signature. They exist in a highly immune-vigilant state, and this profile is further exacerbated with ageing (86). In addition, microglia in the cerebellum are enriched in genes associated with active cell clearance (93), and cerebellar microglia from *Atm^–/–^* mice and individuals with A–T are highly activated as compared to other brain regions (13,33). We therefore propose that persistent DNA damage associated with the loss of ATM might exacerbate the phagocytic abilities of highly reactive cerebellar microglia *in vivo*, resulting in excessive clearance of neuronal material that may contribute to neurodegeneration in A–T.

## DATA AVAILABILITY

Further information and requests for resources and reagents should be directed to and will be fulfilled by the corresponding author.

## Supplementary Material

gkac104_Supplemental_File

## Notes

Present address: Julie Bourseguin, Université d’Orléans, Orléans, France; Centre de Biophysique Moléculaire, Orléans, France.
