# Supplementary material for: Persistent DNA damage associated with ATM kinase deficiency promotes microglial dysfunction
Source: Nucleic Acids Res. 2022 Feb 25;50(5):2700–18. doi: 10.1093/nar/gkac104 (PMC8934660; doi:10.1093/nar/gkac104)
Supplement: gkac104_Supplemental_File [file gkac104_supplemental_file.pdf]

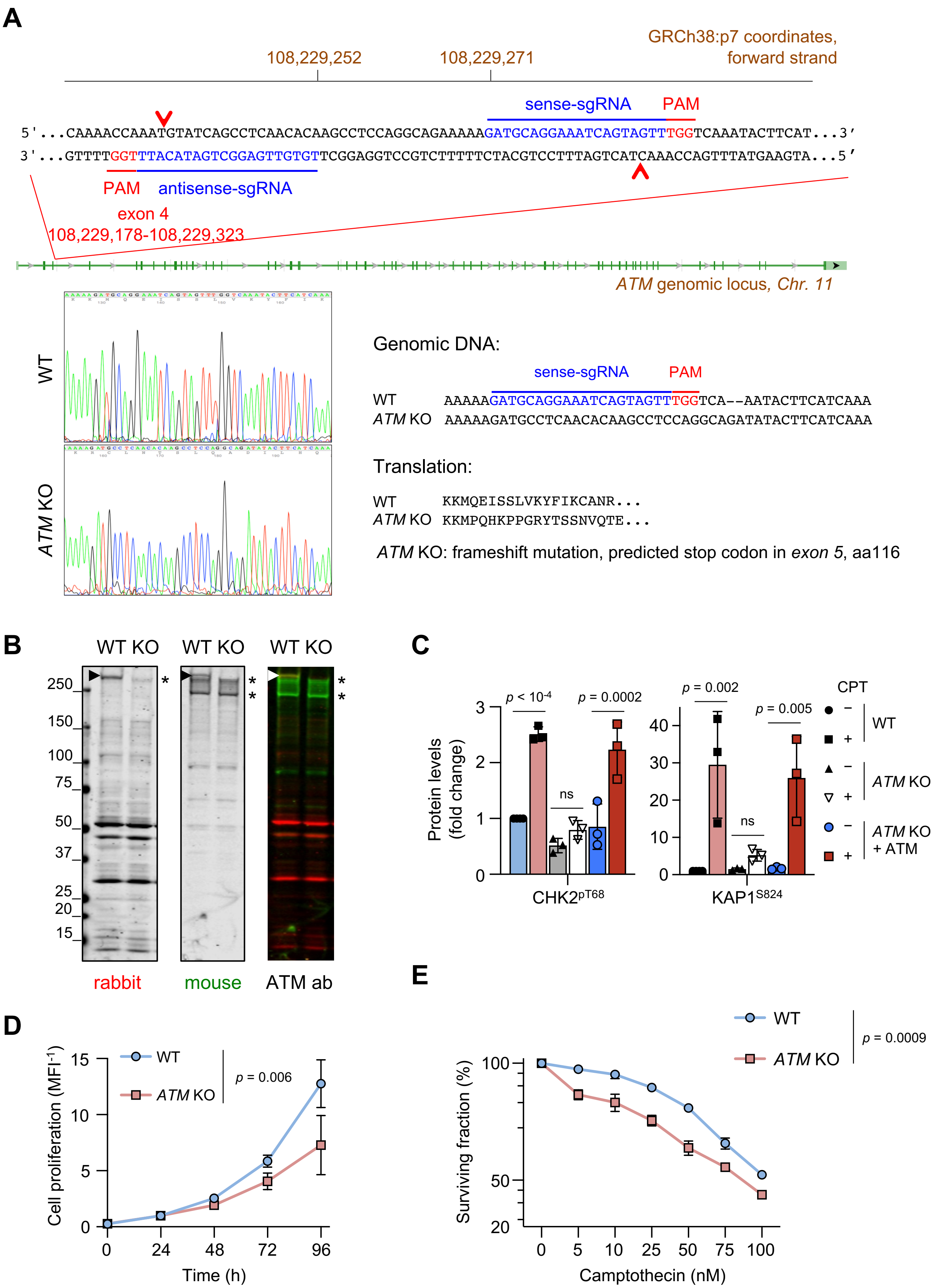
A
GRCh38:p7 coordinates, forward strand
108,229,252
108,229,271
sense-sgRNA
PAM
5'...CAAAACCAAATGTATCAGCCTCAACACAAGCCTCCAGGCAGAAAAAGATGCAGGAAATCAGTAGTTTGGTCAAATACTTCAT...3'
3'...GTTTTGGTTTACATAGTCGGAGTTGTGTTCGGAGGTCCGTCTTTTTCTACGTCCTTTAGTCATCAAACCAGTTTATGAAGTA...5'
PAM
antisense-sgRNA
exon 4
108,229,178-108,229,323
ATM genomic locus, Chr. 11
WT
ATM KO
Genomic DNA:
sense-sgRNA
PAM
WT AAAAAGATGCAGGAAATCAGTAGTTTGGTCA--AATACTTCATCAAA
ATM KO AAAAAGATGCCTCAACACAAGCCTCCAGGCAGATATACTTCATCAAA
Translation:
WT KKMQEISSLVKYFIKCANR...
ATM KO KKMPQHKPPGRYTSSNVQTE...
ATM KO: frameshift mutation, predicted stop codon in exon 5, aa116
B
WT KO
WT KO
WT KO
250
150
100
75
50
37
25
20
15
rabbit
mouse
ATM ab
C
Protein levels (fold change)
p < 10-4
p = 0.0002
ns
p = 0.002
p = 0.005
ns
CHK2pT68
KAP1S824
CPT
WT
ATM KO
ATM KO + ATM
D
Cell proliferation (MFI-1)
WT
ATM KO
p = 0.006
Time (h)
0
24
48
72
96
E
WT
ATM KO
p = 0.0009
Surviving fraction (%)
100
50
20
Camptothecin (nM)
0
5
10
25
50
75
100

Supplementary Figure S1: Generation and characterisation of *ATM* KO HMC3 microglia.
A Design of CRISPR/Cas9-mediated targeting of the *ATM* locus (top). Sanger sequencing of *ATM* KO HMC3 showing 2 bp deletion in *ATM* exon 4 leading to a frame shift, premature STOP codon in exon 5 and generation of a truncated protein. WT HMC3 shown as control.
B Representative immunoblot analysis of ATM protein expression in *ATM* KO vs WT HMC3. Two different ATM antibodies raised against peptides corresponding to residues 1967-1988 (mouse monoclonal, green) and 2250-2600 (rabbit polyclonal, red) in human ATM were used. *Non-specific band.
C Quantification of phosphorylated protein levels as in **Figure 1A**. Data are relative to WT HMC3. Quantification is relative to total protein levels and the loading control. Mean ± S.D. shown (n = 3). Two-way ANOVA with Sidak's multiple comparison's test used.
D Proliferation curves of WT and *ATM* KO HMC3 cells. Proliferation is relative to WT HMC3 at 24 h. Mean ± S.D. shown (n = 3). Paired *t*-test of area under the curve used.
E Sensitivity of WT and *ATM* KO HMC3 cells to camptothecin. Mean ± S.D. shown (n = 3 technical replicates). Paired *t*-test of area under the curve used.

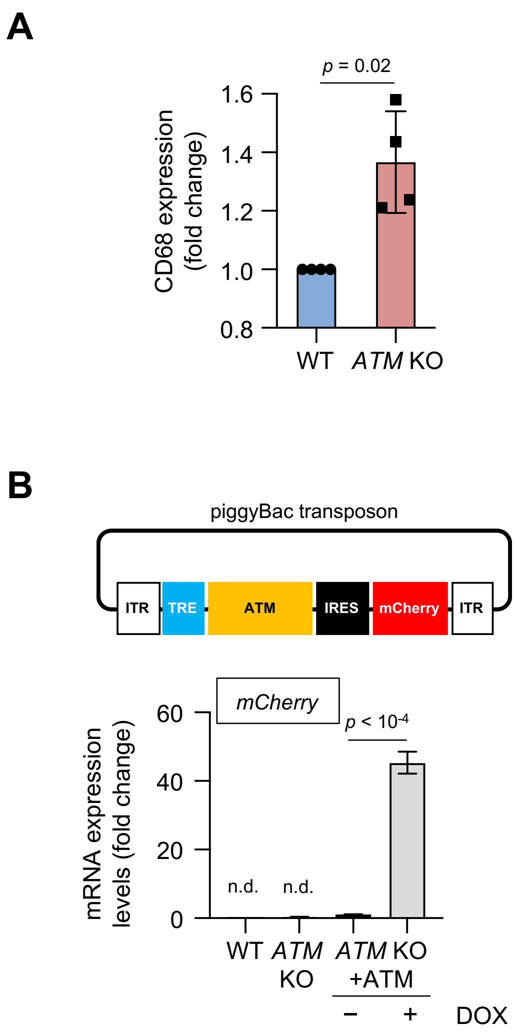

Supplementary Figure S2: *ATM* knockout microglia are activated.

A Quantification of CD68 expression in WT and *ATM* KO HMC3 cells using immunofluorescence. Confocal Z-stack compression images were used for quantification. Mean ± S.D. shown (n = 4). One-sample *t*-test used.

B Schematic of piggyBac transposon carrying *ATM* gene (top) and quantitative RT-qPCR analysis of *mCherry* expression in cells as in **Figure 1A** (bottom). *ATM* KO cells, in which ATM was re-expressed from doxycycline-inducible piggyBac transgene, were cultured in the presence (0.1 µg/mL) or absence of doxycycline (DOX). Expression is relative to *ATM* KO HMC3, no DOX. Reference gene: *IPO8*. Mean ± S.D. shown (n = 3 technical replicates). One sample *t*-test used. N.d.: not detected.

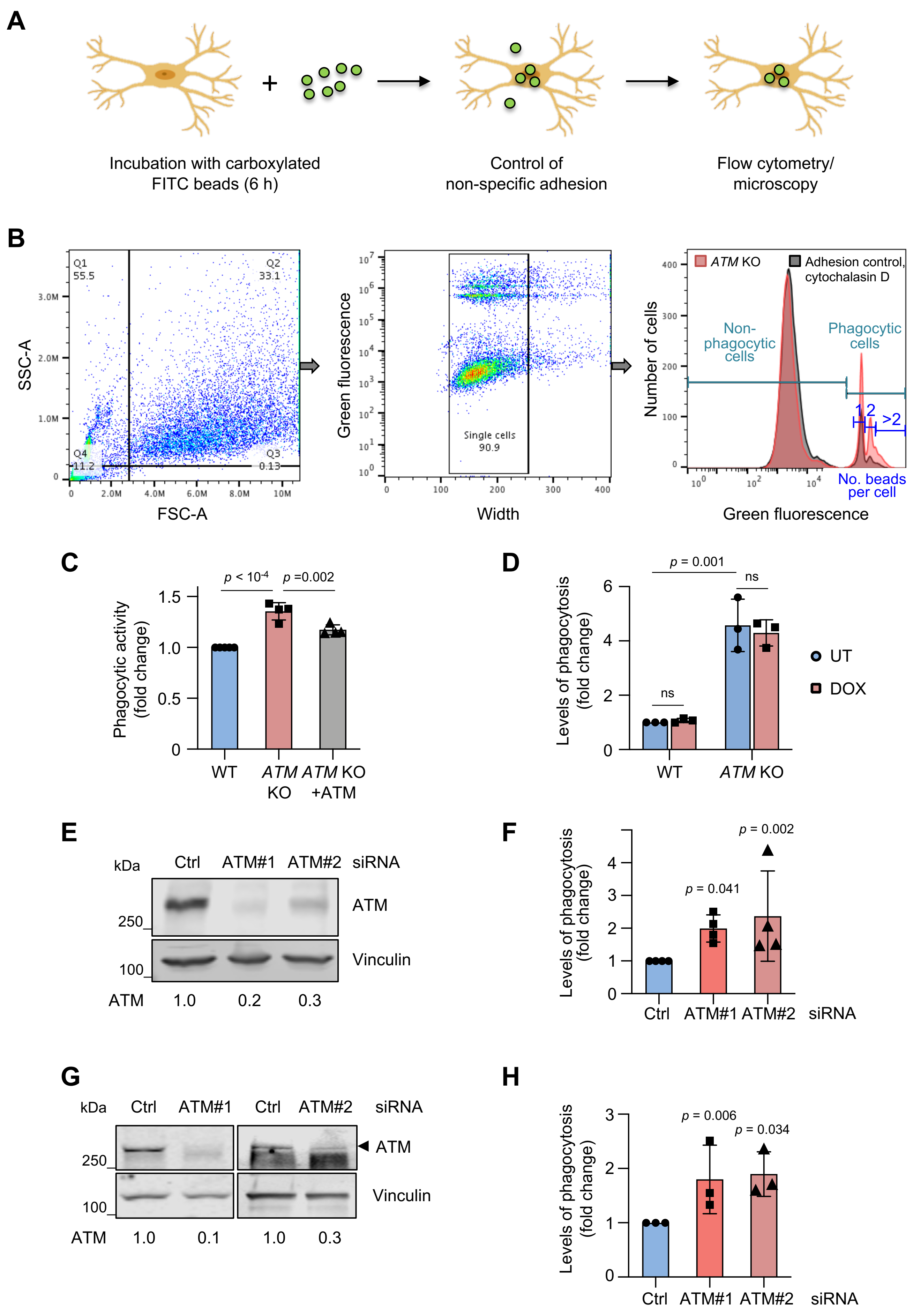
A
Incubation with carboxylated FITC beads (6 h)
Control of non-specific adhesion
Flow cytometry/ microscopy
B
Q1 55.5
Q2 33.1
Q4 11.2
Q3 0.13
SSC-A
FSC-A
Green fluorescence
Width
Single cells 90.9
ATM KO
Adhesion control, cytochalasin D
Non-phagocytic cells
Phagocytic cells
Number of cells
1 2 >2
No. beads per cell
Green fluorescence
C
Phagocytic activity (fold change)
p < 10-4
p =0.002
WT
ATM KO
ATM KO +ATM
D
Levels of phagocytosis (fold change)
p = 0.001
ns
ns
WT
ATM KO
UT
DOX
E
kDa
Ctrl
ATM#1
ATM#2
siRNA
250
100
ATM
Vinculin
ATM 1.0 0.2 0.3
F
Levels of phagocytosis (fold change)
p = 0.002
p = 0.041
Ctrl
ATM#1
ATM#2
siRNA
G
kDa
Ctrl
ATM#1
Ctrl
ATM#2
siRNA
250
100
ATM
Vinculin
ATM 1.0 0.1 1.0 0.3
H
Levels of phagocytosis (fold change)
p = 0.006
p = 0.034
Ctrl
ATM#1
ATM#2
siRNA

Supplementary Figure S3: ATM-deficient microglia are activated.

A Schematic of phagocytosis assays.

B Workflow of phagocytosis analyses using 5 µm beads as substrates by flow cytometry. To control for non-specific adhesion, phagocytosis assays were carried out in the absence or presence of 1 µM cytochalasin D. Percentages of phagocytic cells further referred to as phagocytosis levels were determined. To calculate the relative average number of beads per cell (further referred to as phagocytic activity), mean fluorescent intensity (MFI) of phagocytic cells after subtracting MFI of the cytochalasin D control was divided by MFI of a single bead.

C Phagocytic (5 µm beads) activity of HMC3 microglia as in **Figure 1G**. Activities are relative to WT HMC3. Mean ± S.D. shown (n = 4). One-way ANOVA with Tukey's multiple comparison's test used.

D Phagocytosis levels (5 µm beads) of WT and *ATM* KO HMC3 cells cultured in the presence (0.1 µg/mL) or absence of DOX. Phagocytosis is relative to WT HMC3, in which 5.6 ± 1.7% of cells are phagocytic. Mean ± S.D. shown (n = 3). Two-way ANOVA test used.

E, G Representative immunoblot analysis of ATM silencing using two independent siRNA sequences (ATM#1 and ATM#2) in HMC3 and C20 microglia, respectively. Loading control: vinculin. Quantification of ATM protein levels is relative to loading control.

F, H Phagocytosis levels (5 µm beads) of ATM-deficient HMC3 and C20 as in (**E, G**), respectively. Phagocytosis is relative to cells treated with control siRNA (Ctrl), in which 2.8 ± 2.5% (HMC3) or 11.5 ± 6.1 % (C20) of cells are phagocytic. Mean ± S.D. shown (n = 4 for HMC3, n = 3 for C20). One-sample *t*-test used.

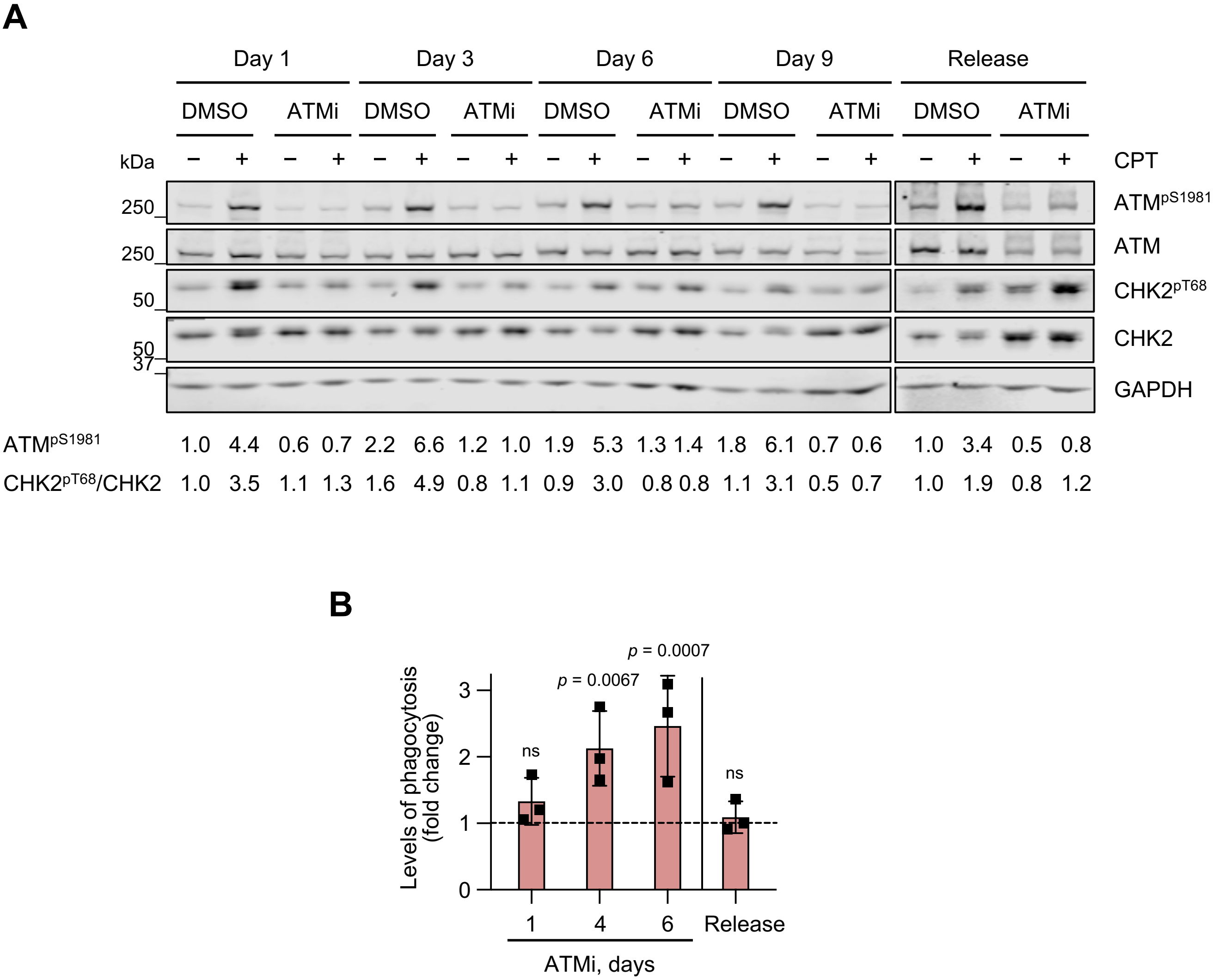

Supplementary Figure S4: ATM inhibition leads to progressively increased phagocytosis.
A Representative immunoblot analysis of the efficacy of long-term inhibition of ATM activity and inhibitor wash out in HMC3 microglia. Cells were treated with DMSO as a control or 10 nM AZD1390 for up to 9 days. The inhibitor was washed out after 6 days of inhibition for an additional 2 days (Release). To test for ATM activity, cells were treated with 1 µM camptothecin (CPT) and analysed at 1 h post-treatment. Quantification of phosphorylated protein levels is relative to total protein levels (except for ATM^{pS1981}) and the loading control.
B Phagocytosis levels (5 µm beads) of HMC3 cells treated with ATM inhibitor as in (**A**). Phagocytosis is relative to WT HMC3 at respective time points (dashed line). Mean ± S.D. shown (n = 3). Two-way ANOVA with Tukey's multiple comparison's test used.

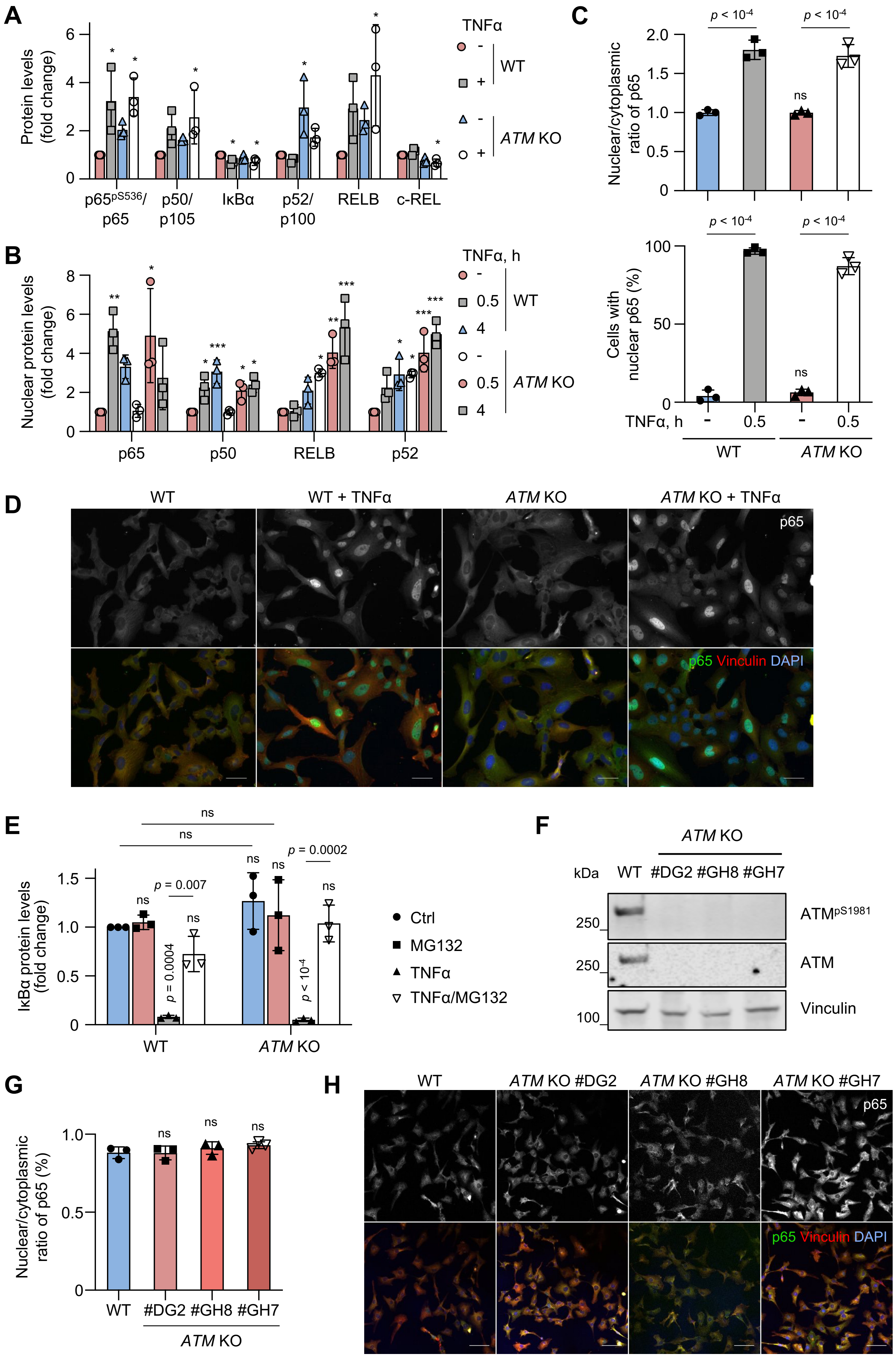
A
Protein levels (fold change)
p65pS536/ p65
p50/ p105
IκBα
p52/ p100
RELB
c-REL
TNFα
WT
ATM KO
B
Nuclear protein levels (fold change)
TNFα, h
p65
p50
RELB
p52
C
Nuclear/cytoplasmic ratio of p65
Cells with nuclear p65 (%)
p < 10-4
ns
TNFα, h
WT
ATM KO
D
WT
WT + TNFα
ATM KO
ATM KO + TNFα
p65
p65 Vinculin DAPI
E
IκBα protein levels (fold change)
ns
p = 0.007
p = 0.0004
p = 0.0002
p < 10-4
Ctrl
MG132
TNFα
TNFα/MG132
WT
ATM KO
F
ATM KO
kDa
WT #DG2 #GH8 #GH7
250
100
ATMpS1981
ATM
Vinculin
G
Nuclear/cytoplasmic ratio of p65 (%)
WT #DG2 #GH8 #GH7
ATM KO
H
WT
ATM KO #DG2
ATM KO #GH8
ATM KO #GH7
p65
p65 Vinculin DAPI

Supplementary Figure S5: The canonical p65-dependent NF-κB pathway is not activated in *ATM* KO microglia under basal conditions.

A Quantification of protein levels as in **Figure 2A**. Data are relative to WT HMC3 and loading control. Mean ± S.D. shown (n = 3). Two-way ANOVA with Dunnet's multiple comparison's test used. *P*-values are shown relative to WT HMC3 (*$p < 0.05$).

B Quantification of nuclear protein levels as in **Figure 2B**. Data are relative to WT HMC3 and loading control. Mean ± S.D. shown (n = 3). Two-way ANOVA with Sidak's multiple comparison's test used *(**$p < 0.05$, **$p < 0.005$, ***$p < 0.0005$*).*

C Quantification of p65 localisation presented as nuclear/cytoplasmic ratio (top) and percentage of cells with nuclear p65 (bottom) in WT and *ATM* KO HMC3 cells. Positive control: 10 µM TNF-α for 30 min. Mean ± S.D. shown (n = 3). Two-way ANOVA with Tukey's multiple comparison's test used.

D Representative images of (**C**). Images in a single Z-plane shown. Scale bar: 50 µm. p65 (green), vinculin (red), DNA (DAPI, blue).

E Quantification of IκBα protein levels as in **Figure 2C**. Data are relative to WT Ctrl. Mean ± S.D. shown (n = 3). Two-way ANOVA with Sidak's multiple comparison's test used (*p*-values are shown relative to WT Ctrl unless specified).

F Representative immunoblot analysis of WT and three clones of *ATM* KO C20 microglia. Loading control: vinculin.

G Quantification of p65 localisation presented as nuclear/cytoplasmic ratio in WT and *ATM* KO C20 cells. Mean ± S.D. shown (n = 3). One-way ANOVA with Dunnett's multiple comparison's test used.

H Representative images of (**G**). Images of compressed z-stacks shown. Scale bar: 50 µm. p65 (green), vinculin (red), DNA (DAPI, blue).

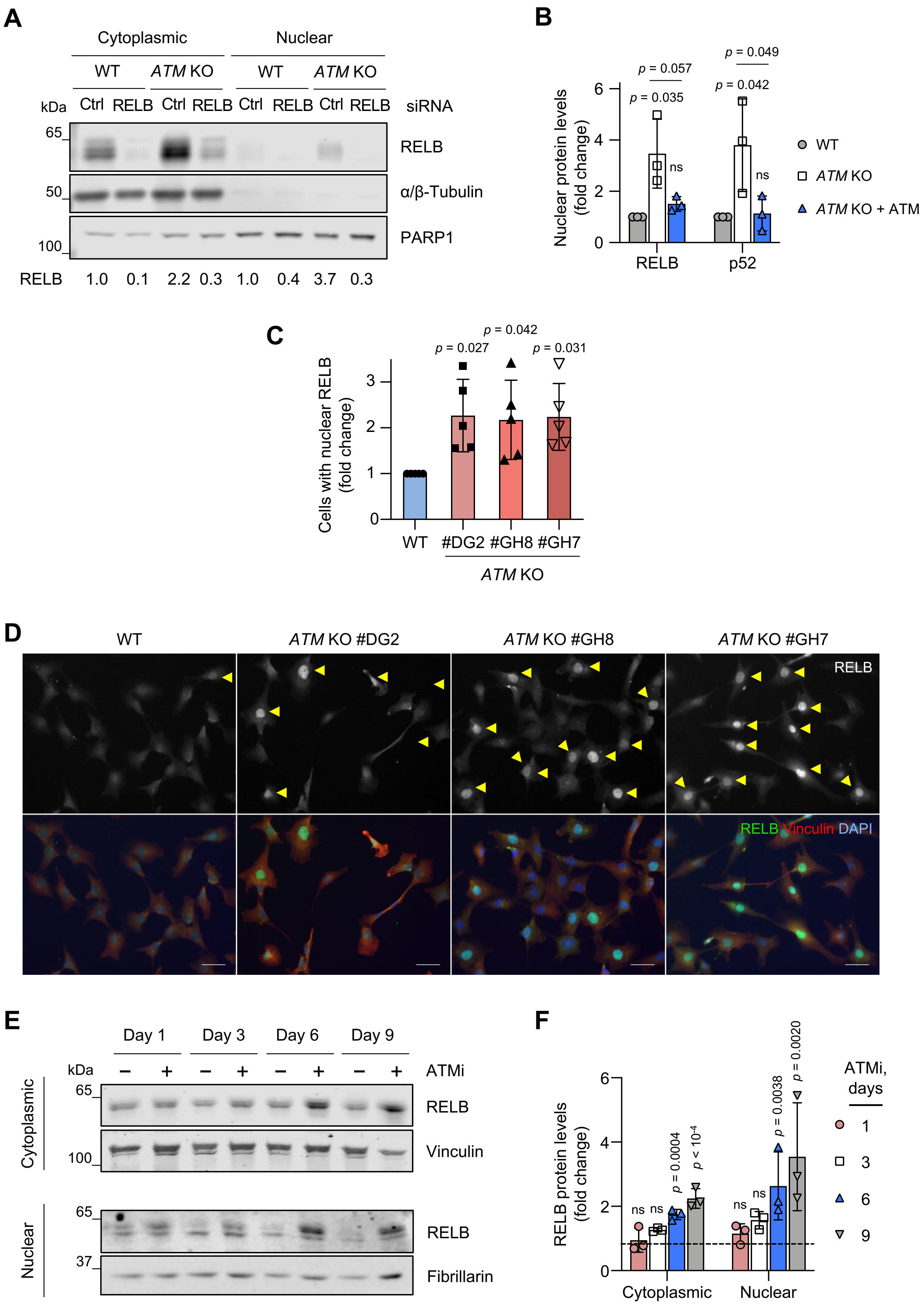
A
Cytoplasmic
Nuclear
WT
ATM KO
WT
ATM KO
kDa
Ctrl RELB Ctrl RELB Ctrl RELB Ctrl RELB
siRNA
65
50
100
RELB
α/β-Tubulin
PARP1
RELB 1.0 0.1 2.2 0.3 1.0 0.4 3.7 0.3
B
Nuclear protein levels (fold change)
p = 0.057
p = 0.035
p = 0.049
p = 0.042
ns
ns
RELB
p52
WT
ATM KO
ATM KO + ATM
C
Cells with nuclear RELB (fold change)
p = 0.027
p = 0.042
p = 0.031
WT
#DG2
#GH8
#GH7
ATM KO
D
WT
ATM KO #DG2
ATM KO #GH8
ATM KO #GH7
RELB
RELB Vinculin DAPI
E
Day 1
Day 3
Day 6
Day 9
kDa
ATMi
Cytoplasmic
Nuclear
65
100
65
37
RELB
Vinculin
RELB
Fibrillarin
F
RELB protein levels (fold change)
ns
ns
p = 0.0004
p < 10⁻⁴
ns
ns
p = 0.0038
p = 0.0020
Cytoplasmic
Nuclear
ATMi, days
1
3
6
9

Supplementary Figure S6: Non-canonical RELB/p52 NF-κB pathway is activated in *ATM* KO microglia.

A Representative immunoblot analysis of siRNA-mediated silencing of RELB in cytoplasmic and nuclear extracts of WT and *ATM* KO HMC3 cells. Loading controls: α/β-tubulin (cytoplasmic), PARP1 (nuclear). Quantification of RELB protein levels is relative to loading control in individual fractions.

B Quantification of nuclear protein levels as in **Figure 2F**. Data are relative to WT HMC3 and loading control. Mean ± S.D. shown (n = 3). One-way ANOVA with Tukey's multiple comparison's test used.

C Quantification of RELB localisation in WT and *ATM* KO C20 cells. Data are relative to WT C20, in which 14.1 ± 5.7% of cells contain nuclear RELB. Mean ± S.D. shown (n = 5). One-way ANOVA with Dunnett's multiple comparison's test used.

D Representative images of (**C**). Images in a single Z-plane shown. Scale bar: 50 µm. RELB (green), vinculin (red), DNA (DAPI, blue).

E Representative immunoblot analysis of RELB in cytoplasmic (left) and nuclear (right) extracts of HMC3 cells treated with 10 nM AZD1390 or DMSO control as in **Supplementary Figure S4A**. Loading controls: vinculin (cytoplasmic), fibrillarin (nuclear).

F Quantification of protein levels as in **(E)**. Data are relative to DMSO control at respective time points (dashed line) and to loading control. Mean ± S.D. shown (n = 3). One-way ANOVA with Holm-Sidak's multiple comparison's test used.

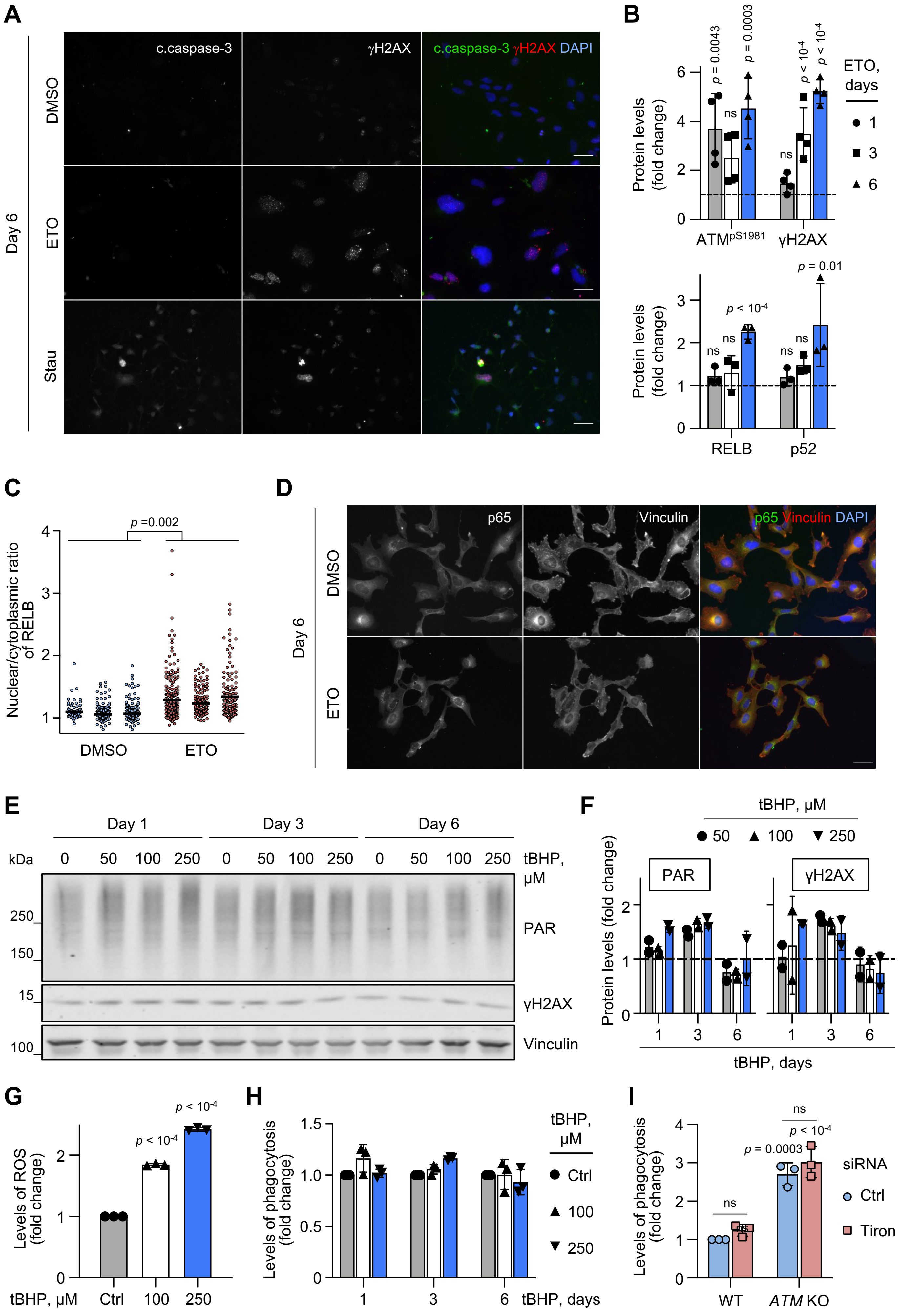

A
c.caspase-3
γH2AX
c.caspase-3 γH2AX DAPI
Day 6
DMSO
ETO
Stau
B
p = 0.0043
ns
p = 0.0003
ns
p < 10-4
p < 10-4
ETO, days
1
3
6
Protein levels (fold change)
ATMpS1981
γH2AX
p = 0.01
p < 10-4
RELB
p52
C
p =0.002
Nuclear/cytoplasmic ratio of RELB
DMSO
ETO
D
p65
Vinculin
p65 Vinculin DAPI
E
Day 1
Day 3
Day 6
kDa
0
50
100
250
tBHP, μM
PAR
γH2AX
Vinculin
250
150
15
100
F
tBHP, μM
50
100
250
PAR
γH2AX
Protein levels (fold change)
tBHP, days
G
Levels of ROS (fold change)
tBHP, μM
Ctrl
p < 10-4
H
Levels of phagocytosis (fold change)
tBHP, μM
tBHP, days
I
siRNA
Ctrl
Tiron
p = 0.0003
WT
ATM KO

Supplementary Figure S7: Persistent DNA damage leads to the activation of RELB/p52 NF-κB pathway.

A Representative immunofluorescence images of HMC3 microglia treated with 0.5 μM etoposide (ETO) or DMSO as a control for 6 days as in **Figure 5E**, and analysed for DNA damage and apoptosis/cell death. Positive control: 0.33 μM staurosporine for 16 h (Stau). Images in a single Z-plane shown. Scale bar: 50 μm. Cleaved caspase-3 (c.caspase-3; green), γH2AX (red), DAPI (blue).

B Quantification of protein levels as in **Figure 5E**. Data are relative to DMSO control at respective time points (dashed line) and to loading control. Mean ± S.D. shown (n = 4 for ATM^{pS1981} and γH2AX, n = 3 for RELB and p52). One-way ANOVA with Tukey's multiple comparison's test used.

C Quantification of RELB nuclear translocation in cells treated as in **Figure 5E**. Control: DMSO. Data presented as nuclear/cytoplasmic ratio of individual cells (100 cells per experiment; n = 3). Nested *t*-test used.

D Representative immunofluorescence images of p65 localisation in cells treated as in **Figure 5E**. Images in a single Z-plane shown. Scale bar: 50 μm. p65 (green), vinculin (red), DAPI (blue).

E Representative immunoblot analysis of DNA damage response markers in WT HMC3 microglia treated with 100-250 μM tert-butyl hydroperoxide (tBHP) for up to 6 days. Loading control: vinculin.

F Quantification of protein levels as in **(E)**. Data are relative to control at respective time points (dashed line) and to loading control. Mean ± S.D. shown (n = 2).

G Levels of intracellular reactive oxygen species (ROS) in cells treated as in **(E)**. ROS levels are relative to WT HMC3. Mean ± S.D. shown (n = 3). One-way ANOVA with Dunnett's multiple comparisons used.

H Phagocytosis levels in WT HMC3 treated as in **(E)**. Phagocytosis is relative to Ctrl at respective time points. Mean ± S.D. shown (n = 3). No significant differences in any of the groups are detected using two-way ANOVA with Dunnett's multiple comparisons.

I Phagocytosis levels in WT and *ATM* KO HMC3 cells treated with 10 μM Tiron for 72 h. Intracellular ROS in *ATM* KO microglia were reduced to (53 ± 19)% compared to untreated Ctrl. Data are relative to WT HMC3. Mean ± S.D. shown (n = 3). Two-way ANOVA with Sidak's multiple comparisons used.

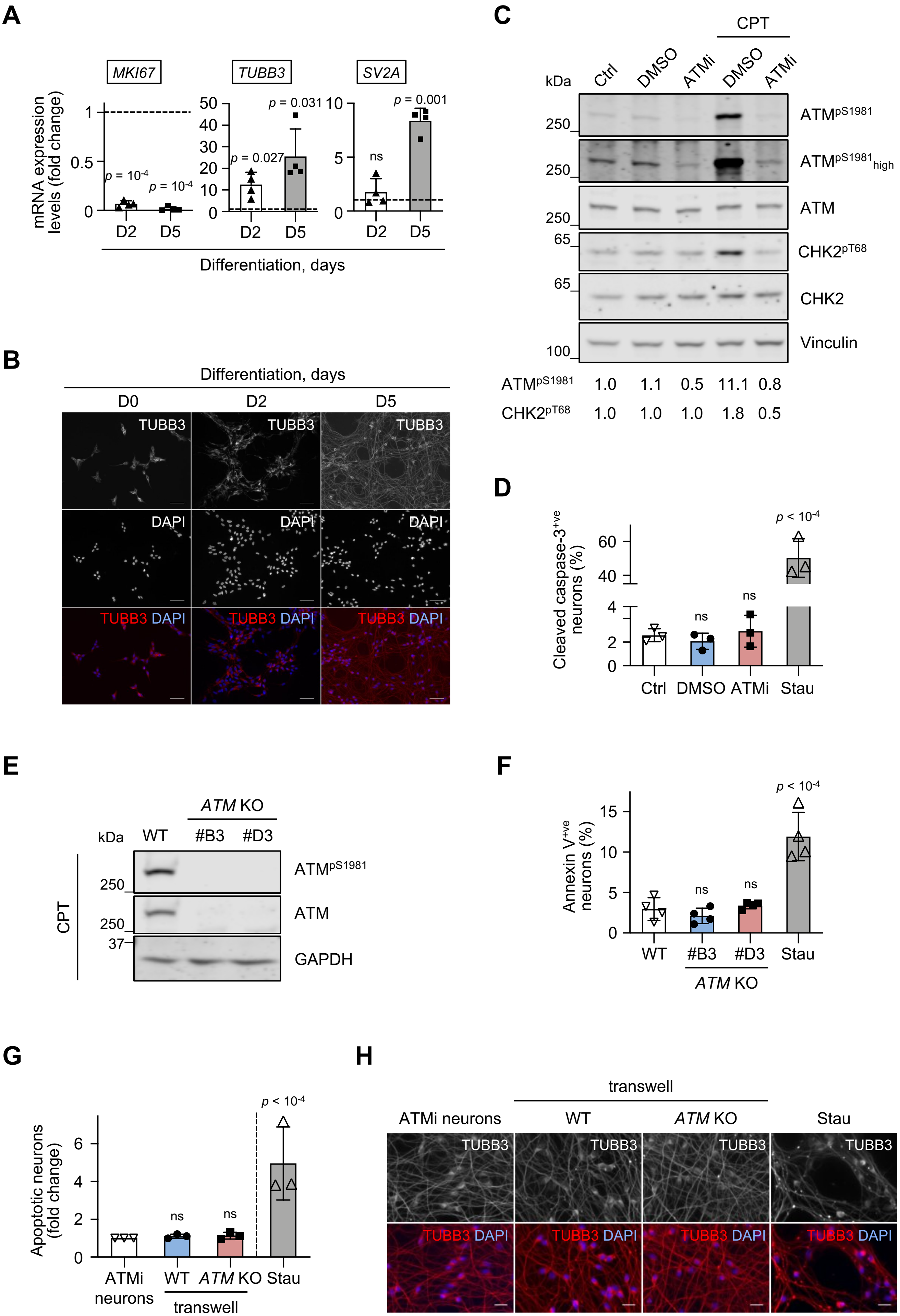

A
MKI67
TUBB3
SV2A
mRNA expression levels (fold change)
p = 10-4
p = 10-4
p = 0.027
p = 0.031
ns
p = 0.001
D2
D5
Differentiation, days
B
Differentiation, days
D0
D2
D5
TUBB3
DAPI
TUBB3 DAPI
C
CPT
kDa
Ctrl
DMSO
ATMi
DMSO
ATMi
ATMpS1981
ATMpS1981high
ATM
CHK2pT68
CHK2
Vinculin
250
65
100
ATMpS1981 1.0 1.1 0.5 11.1 0.8
CHK2pT68 1.0 1.0 1.0 1.8 0.5
D
Cleaved caspase-3+ve neurons (%)
p < 10-4
ns
Ctrl
DMSO
ATMi
Stau
E
ATM KO
kDa
WT
#B3
#D3
CPT
ATMpS1981
ATM
GAPDH
250
37
F
Annexin V+ve neurons (%)
p < 10-4
ns
WT
#B3
#D3
Stau
ATM KO
G
Apoptotic neurons (fold change)
p < 10-4
ns
ATMi neurons
WT
ATM KO
Stau
transwell
H
transwell
ATMi neurons
WT
ATM KO
Stau
TUBB3
TUBB3 DAPI

Supplementary Figure S8: Generation and characterisation of ATM-deficient neuronal cell models.

A RT-qPCR analysis of proliferation MKI67, committed neuronal β3-tubulin (TUBB3) and the synaptic marker SV2A in LUHMES cells at differentiation days 2 and 5 (D2, D5). Expression is relative to day 0 (dashed line). Reference gene: GAPDH. Mean ± S.D. shown (n = 4). Unpaired *t*-test used.

B Representative immunofluorescence images of cells in (**A**). Images in a single Z-plane shown. Scale bar: 50 µm. β3-tubulin (TUBB3; red), DAPI (blue).

C Representative immunoblot analysis showing the efficacy and the persistence of ATM activity inhibition in post-mitotic LUHMES neurons. From day 2, cells were differentiated in the presence of 100 nM AZD1390 or DMSO for 3 days. Untreated post-mitotic neurons were used as a control (Ctrl). After 3 days of inhibition, AZD1390 was washed out and cells were cultured in standard differentiation medium for an additional 48 h. To test for ATM activity, cells were treated with 1 µM camptothecin (CPT) and analysed at 1 h post treatment. Quantification of phosphorylated protein levels is relative to total protein levels (except for ATM^{pS1981}) and the loading control.

D Quantification of cleaved caspase-3 positive LUHMES neurons in post-mitotic LUHMES neurons treated with AZD1390 or DMSO for 3 days during differentiation. Ctrl: standard differentiation medium. Positive control: 0.33 µM staurosporine for 16 h (Stau). Data are relative to Ctrl. Mean ± S.D. shown (n = 3 for Ctrl). One-way ANOVA with Dunnett’s multiple comparison’s test used.

E Representative immunoblot analysis of WT and two clonal *ATM* KO LUHMES cell lines treated with 1 µM camptothecin (CPT) for 1 h. Loading control: GAPDH.

F Quantification of early apoptotic (Annexin V^{+ve}) post-mitotic WT and *ATM* KO LUHMES neurons. Positive control: 1 µM staurosporine for 6 h (Stau). Percentages of late apoptotic (Annexin V^{+ve}/propidium $iodide^{+ve}$) and necrotic (propidium $iodide^{+ve}$) cells were below 1% and similar in all samples. Mean ± S.D. shown (n = 4). One-way ANOVA with Dunnett’s multiple comparison’s test used.

G Immunofluorescence-based quantification of apoptotic (pyknotic and fragmented nuclei) post-mitotic ATM-deficient LUHMES neurons treated as in (**C**) and cultured in a transwell insert in the absence (Ctrl) or presence of WT or ATM KO HMC3 microglia for 48 h. Positive control: 0.33 µM staurosporine for 16 h (Stau). Data are relative to Ctrl, in which 9.1 ± 4.2% of cells are apoptotic. Mean ± S.D. shown (n = 3, two Stau data points are from separate experiments). One-way ANOVA with Dunnett’s multiple comparison’s test used.

H Representative immunofluorescence images of LUHMES cells in (**E**). Images in a single Z-plane shown. Scale bar: 20 µm. β3-tubulin (TUBB3; red), DAPI (blue).

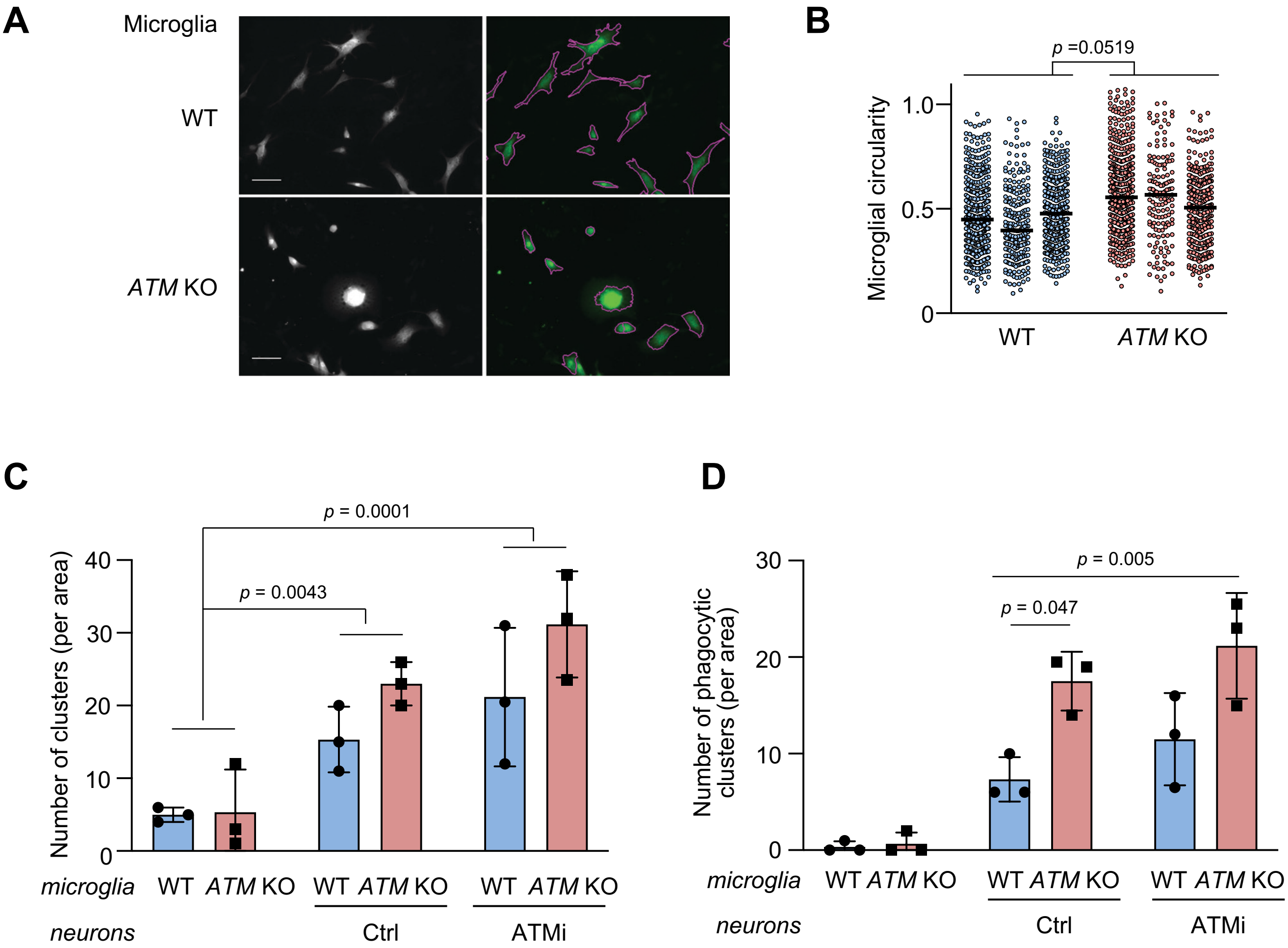

Supplementary Figure S9: ATM knockout neurons aberrantly engulf neurites.

A Representative images of WT and *ATM* KO HMC3 microglia co-cultured with *ATM* KO (clone #D3) post-mitotic LUHMES neurons for 24 h. CFSE-stained microglia in non-clustered areas with segmentation of cells using CellProfiler are shown. Images are in a single Z-plane. Scale bar: 50 µm.

B Quantification of morphological changes in microglia as in (**A**). Data presented as circularity of individual cells (n = 3; minimum 150 cells per experiment). Nested *t*-test used.

C Quantification of clusters formed by WT or *ATM* KO HMC3 microglia on their own or in co-culture with post-mitotic ATM-inhibited LUHMES neurons that were pretreated with AZD1390 or left untreated (Ctrl) as in **Figure 6G**. Mean ± S.D. shown (n = 3). Two-way ANOVA with Sidak's multiple comparison's test used.

D Quantification of phagocytic microglial clusters from (**C**). Mean ± S.D. shown (n = 3). Two-way ANOVA with Tukey's multiple comparison's test used.

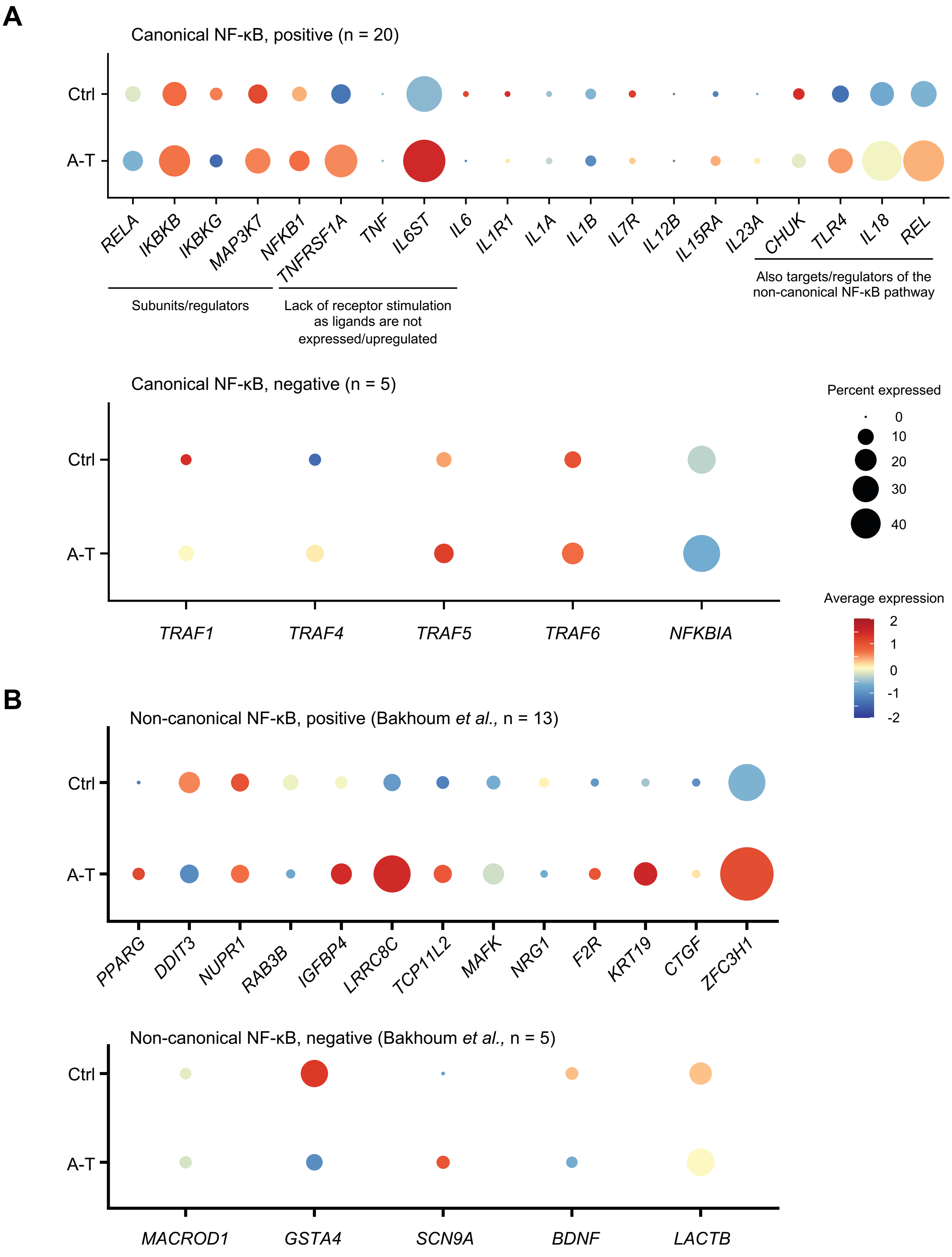

Supplementary Figure S10: Canonical NF-κB is not activated in cerebellar microglia of individuals with A-T.

A, B Dotplot of the average expression and percent cells expressed for individual genes involved in activation (positive) and inhibition (negative) of the (**A**) canonical and (**B**) non-canonical NF-κB pathway in A-T *vs* Ctrl cerebellar microglia. Average expression is represented by the z-score calculated by centering and scaling across microglia from A-T and Ctrl in cerebellum and prefrontal cortex.

Supplementary Table S1. Reagents and tools used in the study.

Reagent/Resource	Reference or Source	Identifier or Catalogue Number
**Experimental Models: Cell lines**		
Human microglial HMC3 cells	Established by Marc Tardieu and kindly provided by Brian Bigger (University of Manchester, UK)	(1)
ATM knockout HMC3 cells	This study	
ATM knockout HMC3 cells expressing pPB-TRE Flag-wtATM	This study	
Human microglial C20 cells	Kind gift of David Alvarez-Carbonell	(2)
ATM knockout C20 cells (clones G:H7, G:H8, D:G2)	This study	
Human mesencephalic LUHMES cells	ATCC	CRL2927
ATM knockout LUHMES cells (clones B3, D3)	This study	
**Bacterial Strains**		
DH5α	Invitrogen	18265017
NEB® 10-beta	New England Biolabs	C3019
**Recombinant DNA**		
pSpCas9n(BB)-2A-Puro (PX462)	Addgene	48141 (3)
AIO-GFP	Addgene	74119 (4)
pSpCas9(BB)-2A-Puro V2.0	Addgene	62988 (3)
pcDNA3.1 Flag-His-ATM plasmid	Addgene	31985 (5)
rtTA	kind gift of Bon-Kyoung Koo	(6)
pPB-transposase	kind gift of Bon-Kyoung Koo	(6)
pPB-TRE IRES-mCherry	kind gift of Bon-Kyoung Koo	(6)
pPB-TRE Flag-wtATM	This paper	
**Oligonucleotides**		
**Cloning**		
ATM fragment 1 Forward:	tcattttggcaaagaattccTAATGGACTACAAGGACG	
ATM fragment 1 Reverse:	aaagggagaaGCTACGTAATGACACATC	
ATM fragment 2 Forward:	attacgtagcTTCTCCCTTTGTTGTGAC	
ATM fragment 2 Reverse:	cgatatcaagcttatcgagcGATCACACCCAAGCTTTC	
**Fragment analysis**		

ATM-exon4 Forward	GCTCTTTGTGATGGCATGAACAGCTTTTG	
ATM-exon4 Forward	CTCACGCGACAGTAATCTGTTAAGCCAT	

**RT-qPCR**		
mCherry Forward	CACGAGTTCGAGATCGAGGG	
mCherry Reverse	CAAGTAGTCGGGATGTCG	
IL6 Forward	AGACAGCCACTCACCTCTTCAG	
IL6 Reverse	TTCTGCCAGTGCCTCTTTGCTG	
IL8 Forward	ATGACTTCCAAGCTGGCCGTGGCT	
IL8 Reverse	TCTCAGCCCTCTTCAAAAACTTCTC	
IL1B Forward	CCACAGACCTTCCAGGAGAATG	
IL1B Reverse	GTGCAGTTCAGTGATCGTACAGG	
TNFA Forward	CAGCCTCTTCTCCTTCCTGAT	
TNFA Reverse	GCCAGAGGGCTGATTAGAGA	
IL4 Forward	ACCATGAGAAGGACACTCGC	
IL4 Reverse	CTCTGGTTGGCTTCCTTCACA	
CCL5 Forward	GCTGTCATCCTCATTGCTACTG	
CCL5 Reverse	TGGTGTAGAAATACTCCTTGATGTG	
MAP3K14 Forward	ACAACGAGGGTGTCCTGCTC	
MAP3K14 Reverse	TCAGCTCCTCTGCCCGAAA	
MKI67 Forward	TGGGCGAAGTTCACAGTCAA	
MKI67 Reverse	TGAGCACTCTGTAGGGTCGA	
TUBB3 Forward	CGCCCAGTATGAGGGAGAT	
TUBB3 Reverse	AGTCGCCCACGTAGTTGC	
SV2A Forward	AGATCGGCAGGCTCAGAAT	
SV2A Reverse	GACAGGAAGAAGCAGGAGACA	
RPS13 Forward	CGAAAGCATCTTGAGAGGAACA	
RPS13 Reverse	TCGAGCCAAACGGTGAATC	
IPO8 Forward	GGCACCACTCAGCGAGGAT	
IPO8 Reverse	TGTTGTTCAATCTTCTTCTTTGCCT	

**siRNA Oligonucleotides**		
siCtrl (negative control)	Eurogentec	SR-CL000-005
siATM#1	GGGCCUUUGUUCUUCGAGACGUUAU	kind gift of Ester Hammond
siATM#2	GAGAGGAGACAGCUUGUUAUU	(7)
siRELB	CACAGAUGAAUUGGAGAUCAU	(8)

si^{pan}NF-κB	AAGGUGCAGAAAGAGGACA	(9)
siNIK	CCTGUGUAGACAGCCAGAAACCCUU	

**Antibodies**		
β-Actin (WB)	Abcam	ab6276
Fibrillarin (WB)	Abcam	ab4566
GAPDH (WB)	Cell Signaling	2118
Lamin A/C	Cell Signaling	2032
α/β-Tubulin (WB)	Cell Signaling	2148
Vinculin (WB, IF)	Abcam	ab18058
ATM (WB)	Abcam	ab78
ATM (WB, IF)	Bethyl Laboratories	A300-299A
ATM phosphoSer1981 (WB, IF)	Abcam	ab81292
Caspase-3 (WB)	Santa Cruz	sc-7272
Cleaved caspase-3 (WB, IF)	Cell Signaling	9664
CD40 (Flow cytometry)	GeneTex	GTX14148
CD68 (IF)	Santa Cruz	sc-20060
KAP1 (WB)	Abcam	ab22553
KAP1 phosphoS824 (WB)	Abcam	ab70369
CHK2 (WB)	Cell Signaling	3440
CHK2 phosphoThr68 (WB)	Cell Signaling	2661
γH2A.X (WB, IF)	Millipore	05-636
IκBα (WB)	Cell Signalling	4814
NIK (WB)	Cell Signaling	4994
PAR (WB)	Trevigen	4335-MC-100
PARP-1 (WB)	kind gift of Grigory Dianov	(10)
β3-tubulin	Santa Cruz	sc-80005
RELA (p65; WB, IF)	Cell Signaling	8242
RELA phosphoSer536 (WB)	Cell Signaling	3033S
RELB (IF)	Abcam	ab180127
RELB (WB)	Cell Signaling	4922
c-REL (WB)	Cell Signaling	12707
p50/p105 (WB)	Cell Signaling	13586
p52/p100 (WB)	Cell Signaling	4882
AlexaFluor 680 goat anti-rabbit (WB)	Molecular Probes	A27042
IR Dye 800 goat anti-mouse (WB)	LI-COR Biosciences	926-32210

AlexaFluor 488 goat anti-mouse (IF, Flow cytometry)	Invitrogen	A11001
AlexaFluor 488 goat anti-rabbit (IF)	Invitrogen	A32731
AlexaFluor 594 goat anti-mouse (IF)	Invitrogen	A-11032
Alexa Fluor 594 goat anti-rabbit (IF)	Abcam	ab150080
**Chemicals, enzymes and other reagents**		
ATM inhibitor AZD1390	Selleckchem	S8680
Camptothecin	Cambridge Bioscience	CAY11694
Etoposide	Cambridge Bioscience	CAY12092
Recombinant TNFα	Preprotech	300-01A
tert-Butyl hydroperoxide	Merck	168521
4,5-dihydroxy-1,3-benzenedisulfonic acid (Tiron)	Merck	172553
Carboxyfluorescein succinimidyl ester (CFSE)	Tonbo Biosciences	13-0850
CM-H2DCF-DA	ThermoFisher	C6827
Puromycin	Santa Cruz	sc-108071
Hygromycin B	Millipore	400051
Doxycycline	Merck	D9891
Phusion High-Fidelity DNA Polymerase	ThermoFisher	F530
Lipofectamine2000	Invitrogen	11668019
Lipofectamine RNAiMax	Invitrogen	13778-150
Polyethylenimine (PEI)	Polysciences Inc.	23966
DNAse I	ThermoFisher	EN0521
Carboxyl Fluorescent Particles, 5.0-5.9 µm	Spherotech	CFH-5052-2
SYBR Gold	Molecular Probes	S11494
**Kits and assays**		
RNeasy Mini Kit	QIAGEN	74106
qPCRBIO cDNA Synthesis Kit	PCR Biosystems	PB30.11-10
qPCRBIO SyGreen Blue Mix Lo-ROX	PCR Biosystems	PB20.15-20
NEBuilder® HiFi DNA Assembly Cloning Kit	New England Biolabs	E5520
Glial-Mag Magnetofection	OzBiosciences	GL00500

CellTrace™ Violet Cell Proliferation Kit (CTV)	ThermoFisher	C34557
CellTiter-Glo® 2.0	Promega	G9243
**Software and Algorithms**		
QuantStudioTM Design & Analysis v1.5.1	ThermoFisher	
Image Studio™ Lite Software	Licor	
Image J	NIH	
FlowJo 10.4.2	FlowJo	
GraphPad Prism	GraphPad Software	

Supplementary Table S2. NF-κB gene set lists.

Non-canonical NF-κB pathway members		Non-canonical NF-κB pathway targets (11)		Canonical NF-κB pathway	
Negative (n=5)	Positive (n=11)	Negative (n=5)	Positive (n=13)	Negative (n=5)	Positive (n=20)
TRAF2	LTBR	MACROD1	PPARG	TRAF1	TNFRSF1A
TRAF3	CD40	GSTA4	DDIT3	TRAF4	IL1R1
BIRC2	TNFRSF8	SCN9A	NUPR1	TRAF5	TLR4
BIRC3	CD27	BDNF	RAB3B	TRAF6	RELA
TRIM9	TNFRSF13C	LACTB	IGFBP4	NFKBIA	REL
FBXW7	TNFRSF1B		LRRC8C		NFKB1
	TNFRSF11A		TCP11L2		CHUK
	RELB		MAFK		IKBKB
	NFKB2		NRG1		IKBKG
	MAP3K14		F2R		MAP3K7
			KRT19		TNF
			CTGF		IL12B
			ZFC3H1		IL15RA
					IL18
					IL1A
					IL1B
					IL23A
					IL6
					IL6ST
					IL7R

Negative regulators/supressed genes are shown in red. n - number of genes in a gene set.

Supplementary Table S3. *T*-score permutation test results with *p*-values for A-T *vs* control (Ctrl) cerebellum as in **Figure 7A**.

Gene set	Observed average *t*-score	Permuted average *t*-score	*p*-value	Bonferroni adjusted *p*-value
Canonical NF-κB, positive (n=20)	1.270	0.217	0.117	0.700
Canonical NF-κB, negative (n=5)	2.954	0.233	0.054	0.323
Non-canonical NF-κB, positive (n=10)	3.532	0.210	0.005	0.027
Non-canonical NF-κB, negative (n=6)	-1.560	0.212	0.862	1.000
Non-canonical NF-κB, positive (11) (n=13)	1.948	0.232	0.053	0.316
Non-canonical NF-κB, negative (11) (n=5)	-2.256	0.242	0.914	1.000

Pathways, enrichment of which is significantly different between A-T *vs* control cerebellar microglia (FDR < 0.05), are highlighted in green. FDR – false discovery rate.

Supplementary Table S4. logFC and *t*-scores for individual genes in A-T *vs* Ctrl cerebellum as in **Figure 7B** and **Supplementary Figure S10**.

Gene set	Gene	Log FC	Log CPM	F-statistic	*p*-value	FDR	*t*-score
Canonical NF-κB, negative (n=5)	TRAF1	0.125	10.889	12.734	0.178	0.696	3.568
	TRAF4	0.314	10.897	5.403	0.321	0.992	2.324
	TRAF5	0.149	10.900	8.040	0.187	0.716	2.835
	TRAF6	0.177	10.908	8.246	0.129	0.566	2.872
	NFKBIA	0.336	10.992	10.054	0.009	0.091	3.171
Canonical NF-κB, positive (n=20)	TNFRSF1A	0.737	11.025	44.182	0.000	2.15E-06	6.647
	IL1R1	-0.026	10.877	0.591	0.925	1.000	-0.768
	TLR4	0.381	10.964	14.274	0.007	0.070	3.778
	RELA	0.131	10.926	2.937	0.372	1.000	1.714
	REL	0.702	11.157	31.385	0.000	1.51E-06	5.602
	NFKB1	0.304	10.926	14.740	0.023	0.170	3.839
	CHUK	-0.071	10.898	0.113	0.816	1.000	-0.337
	IKBKB	0.177	11.019	3.217	0.126	0.556	1.793
	IKBKG	-0.116	10.895	7.121	0.392	1.000	-2.669
	MAP3K7	0.186	10.960	5.109	0.111	0.514	2.260
	TNF	0.000	10.876	0.000	1.000	1.000	0.000
	IL12B	0.000	10.876	0.000	1.000	1.000	0.000
	IL15RA	0.125	10.885	28.547	0.121	0.542	5.343
	IL18	0.892	11.150	46.178	0.000	1.83E-09	6.795
	IL1A	-0.001	10.878	3.382	0.676	1.000	-1.839
	IL1B	-0.178	10.892	27.403	0.043	0.266	-5.235
	IL23A	0.017	10.877	46.194	0.185	0.712	6.797
	IL6	-0.009	10.876	10.596	0.215	0.781	-3.255
	IL6ST	0.565	11.310	22.090	0.000	1.03E-04	4.700
	IL7R	-0.297	10.887	95.257	0.001	0.019	-9.760
Non-canonical NF-κB, negative (11) (n=5)	MACROD1	-0.046	10.883	1.318	0.808	1.000	-1.148
	GSTA4	-0.799	10.906	139.915	0.000	2.37E-05	-11.829
	SCN9A	0.128	10.882	52.211	0.029	0.202	7.226
	BDNF	-0.199	10.883	36.573	0.622	1.000	-6.048
	LACTB	0.024	10.931	0.268	0.687	1.000	0.518
Non-canonical NF-κB, positive (11) (n=13)	PPARG	0.060	10.881	46.643	0.065	0.359	6.830
	DDIT3	-0.211	10.902	17.998	0.082	0.420	-4.242
	NUPR1	0.318	10.906	0.781	0.406	1.000	0.884
	RAB3B	-0.226	10.881	196.329	0.001	0.021	-14.012
	IGFBP4	0.516	10.924	35.241	0.007	0.074	5.936
	LRRC8C	0.776	10.980	52.088	0.000	3.46E-06	7.217
	TCP11L2	0.071	10.893	7.730	0.291	0.935	2.780
	MAFK	0.285	10.903	27.120	0.011	0.104	5.208
	NRG1	-0.085	10.878	89.556	0.111	0.515	-9.463
	F2R	0.062	10.882	12.100	0.361	1.000	3.478
	KRT19	1.395	11.112	238.154	0.000	7.45E-15	15.432

	CTGF	0.050	10.883	0.123	0.875	1.000	0.350
	ZFC3H1	0.562	11.129	24.219	0.000	6.16E-05	4.921
Non-canonical NF-κB, negative (n=6)	TRAF2	0.146	10.889	1.566	0.671	1.000	1.251
	TRAF3	0.111	10.910	3.733	0.337	1.000	1.932
	BIRC2	0.223	10.979	6.197	0.055	0.318	2.489
	BIRC3	0.081	10.888	2.421	0.543	1.000	1.556
	TRIM9	-1.241	11.007	116.771	0.000	1.14E-10	-10.806
	FBXW7	-0.691	11.012	33.434	0.000	1.76E-05	-5.782
Non-canonical NF-κB, positive (n=10)	LTBR	0.829	11.000	58.181	0.000	2.27E-07	7.628
	CD40	-0.135	10.897	4.545	0.358	1.000	-2.132
	TNFRSF8	0.044	10.878	22.773	0.357	1.000	4.772
	CD27	0.000	10.876	0.000	1.000	1.000	0.000
	TNFRSF13C	0.073	10.934	0.800	0.558	1.000	0.895
	TNFRSF1B	0.785	11.081	41.537	0.000	6.20E-07	6.445
	TNFRSF11A	0.173	10.902	12.747	0.093	0.457	3.570
	RELB	0.216	10.886	58.181	0.015	0.124	7.628
	NFKB2	0.098	10.894	6.464	0.286	0.924	2.542
	MAP3K14	0.236	10.906	15.740	0.047	0.282	3.967

Genes, expression of which is significantly different between A-T and Ctrl (FDR < 0.05), are highlighted in green. FC – fold change, CPM - counts per million, and FDR – false discovery rate.

SUPPLEMENTARY REFERENCES

1. Janabi, N., Peudenier, S., Heron, B., Ng, K.H. and Tardieu, M. (1995) Establishment of human microglial cell lines after transfection of primary cultures of embryonic microglial cells with the SV40 large T antigen. *Neurosci Lett*, **195**, 105-108.
2. Garcia-Mesa, Y., Jay, T.R., Checkley, M.A., Luttge, B., Dobrowolski, C., Valadkhan, S., Landreth, G.E., Karn, J. and Alvarez-Carbonell, D. (2017) Immortalization of primary microglia: a new platform to study HIV regulation in the central nervous system. *Journal of NeuroVirology*, **23**, 47-66.
3. Ran, F.A., Hsu, P.D., Wright, J., Agarwala, V., Scott, D.A. and Zhang, F. (2013) Genome engineering using the CRISPR-Cas9 system. *Nature Protocols*, **8**, 2281-2308.
4. Chiang, T.W.W., Le Sage, C., Larrieu, D., Demir, M. and Jackson, S.P. (2016) CRISPR-Cas9D10A nickase-based genotypic and phenotypic screening to enhance genome editing. *Scientific Reports*, **6**, 1-17.
5. Canman, C.E., Lim, D.S., Cimprich, K.A., Taya, Y., Tamai, K., Sakaguchi, K., Appella, E., Kastan, M.B. and Siliciano, J.D. (1998) Activation of the ATM kinase by ionizing radiation and phosphorylation of p53. *Science*, **281**, 1677-1679.
6. Andersson-Rolf, A., Mustata, R.C., Merenda, A., Kim, J., Perera, S., Grego, T., Andrews, K., Tremble, K., Silva, J.C., Fink, J. *et al.* (2017) One-step generation of conditional and reversible gene knockouts. *Nat Methods*, **14**, 287-289.
7. Berkovich, E., Monnat, R.J., Jr. and Kastan, M.B. (2007) Roles of ATM and NBS1 in chromatin structure modulation and DNA double-strand break repair. *Nat Cell Biol*, **9**, 683-690.
8. Newman, A.C., Kemp, A.J., Drabsch, Y., Behrends, C. and Wilkinson, S. (2017) Autophagy acts through TRAF3 and RELB to regulate gene expression via antagonism of SMAD proteins. *Nat Commun*, **8**, 1537.
9. van Uden, P., Kenneth, N.S., Webster, R., Muller, H.A., Mudie, S. and Rocha, S. (2011) Evolutionary conserved regulation of HIF-1beta by NF-kappaB. *PLoS Genet*, **7**, e1001285.
10. Woodhouse, B.C., Dianova, II, Parsons, J.L. and Dianov, G.L. (2008) Poly(ADP-ribose) polymerase-1 modulates DNA repair capacity and prevents formation of DNA double strand breaks. *DNA Repair (Amst)*, **7**, 932-940.
11. Bakhoum, S.F., Ngo, B., Laughney, A.M., Cavallo, J.A., Murphy, C.J., Ly, P., Shah, P., Sriram, R.K., Watkins, T.B.K., Taunk, N.K. *et al.* (2018) Chromosomal instability drives metastasis through a cytosolic DNA response. *Nature*, **553**, 467-472.
